# Influence of nuclear dynamics on molecular attosecond photoelectron interferometry

**DOI:** 10.1126/sciadv.adh7747

**Published:** 2023-09-30

**Authors:** Dominik Ertel, David Busto, Ioannis Makos, Marvin Schmoll, Jakub Benda, Hamed Ahmadi, Matteo Moioli, Fabio Frassetto, Luca Poletto, Claus Dieter Schröter, Thomas Pfeifer, Robert Moshammer, Zdeněk Mašín, Serguei Patchkovskii, Giuseppe Sansone

**Affiliations:** ^1^ Physikalisches Institut, Albert-Ludwigs-Universität Freiburg, Hermann-Herder-Straße 3, 79104 Freiburg, Germany.; ^2^ Department of Physics, Lund University, PO Box 118, SE-221 00 Lund, Sweden.; ^3^ Institute of Theoretical Physics, Faculty of Mathematics and Physics, Charles University, V Holešovǐkách 2, 180 00, Prague 8, Czech Republic.; ^4^ Istituto di Fotonica e Nanotecnologie, CNR, 35131 Padova, Italy.; ^5^ Max-Planck-Institut für Kernphysik, 69117 Heidelberg, Germany.; ^6^ Max Born Institute, Max-Born-Str. 2A, D-12489 Berlin, Germany.

## Abstract

In extreme ultraviolet spectroscopy, the photoionization process occurring in a molecule due to the absorption of a single photon can trigger an ultrafast nuclear motion in the cation. Taking advantage of attosecond photoelectron interferometry, where the absorption of the extreme ultraviolet photon is accompanied by the exchange of an additional infrared quantum of light, one can investigate the influence of nuclear dynamics by monitoring the characteristics of the photoelectron spectra generated by the two-color field. Here, we show that attosecond photoelectron interferometry is sensitive to the nuclear response by measuring the two-color photoionization spectra in a mixture of methane (CH_4_) and deuteromethane (CD_4_). The effect of the different nuclear evolution in the two isotopologues manifests itself in the modification of the amplitude and contrast of the oscillations of the photoelectron peaks. Our work indicates that nuclear dynamics can affect the coherence properties of the electronic wave packet emitted by photoionization on a time scale as short as a few femtoseconds.

## Teaser

Nuclear dynamics can affect the molecular photoionization process in two-color fields.

## INTRODUCTION

Attosecond and strong-field spectroscopy have been widely used for the investigation of correlated electronic dynamics in atoms (*1*) and correlated electronic-nuclear dynamics in molecules (*2*). In atoms, strong-field photoionization can create a coherent superposition of electronic states of the cation, leading to electronic charge oscillations that have been resolved in time by attosecond time-resolved transient absorption (*3*). In molecules, it was shown that the process of tunneling ionization launches correlated electronic-nuclear dynamics (*4*), offering the possibility to use the electronic wave packet recolliding with the parent cation for imaging molecular structures (*5*) and ultrafast nuclear dynamics (*6*). The latter is expected to play a crucial role in the observation of electronic processes in complex molecules (*7*, *8*).

An approach for studying the effect of nuclear motion on correlated electronic-nuclear dynamics is the investigation of the response of molecules presenting the same structure and chemical composition but with isotopic substitution of one or more constituents. Under these conditions, the chemical properties of the systems, i.e., their electronic properties, are typically not notably affected, giving the possibility to isolate the effect of the different time scales of the nuclear response in the isotopologues. Isotopic effects in high-order harmonic generation (HHG) have been first predicted (*9*) and then experimentally observed in hydrogen, deuterium, and methane (*10*, *11*). Nuclear-motion effects in HHG appear to be universal (*12*) and have been demonstrated in molecules as large as toluene (*13*). Recently, different decay times in the relaxation dynamics of the two isotopologues $C_{2}H_{4}$ and $C_{2}D_{4}$ have been observed in extreme ultraviolet (XUV)-infrared (IR) pump-probe experiments (*14*). Last, the relevance of isotopic effects in methane (${\mathrm{CH}}_{4}$) and deuteromethane (${\mathrm{CD}}_{4}$) in the reorganization of the molecular structure of the cation after sudden ionization (*15*) was investigated theoretically (*16*).

In attosecond photoelectron spectroscopy, the reconstruction of attosecond beating by interference of two-photon transitions (RABBIT) technique (*17*) has been widely used first for attosecond metrology applications and, later, for the investigation of attosecond electronic dynamics in all states of matter (*18*). In particular, RABBIT measurements in molecules have evidenced the role played on photoionization delays by shape resonances (*19*, *20*) and by the emission direction of the outgoing photoelectron (*21*, *22*). Moreover, the effect of the vibrational degrees of freedom and nuclear dynamics on the photoionization phases was observed in nitrogen (*23*) and hydrogen (*24*, *25*).

Methane and deuteromethane are ideal candidates for the investigation of coupled electronic-nuclear dynamics on an ultrashort time scale due to ultrafast nonadiabatic dynamics triggered by the photoionization process. It manifests itself in the coupling between degenerate electronic and vibrational degrees of freedom, resulting in reduced symmetry in the configuration of the molecular cation (*26*). Exploiting the correlated dynamics of the electron-nuclear wave packet launched by tunneling ionization in an intense IR field, a different efficiency of the HHG process driven by the same intense IR field in these isotopologues was observed (*10*).

Here, we characterize the effect of nuclear dynamics in attosecond photoelectron interferometry by measuring, under the same experimental conditions, the photoelectron spectrograms generated in methane and its deuterated counterpart combining an attosecond pulse train and a synchronized IR field. The experimental data evidence an effect on the amplitude and on the contrast of the oscillations of the photoelectron yield generated in the two-color photoionization process. The experimental data can be interpreted considering the differences in the nuclear autocorrelation functions and in the extension of the vibrational ground states in the two molecules.

## RESULTS

**Fig. 1.**
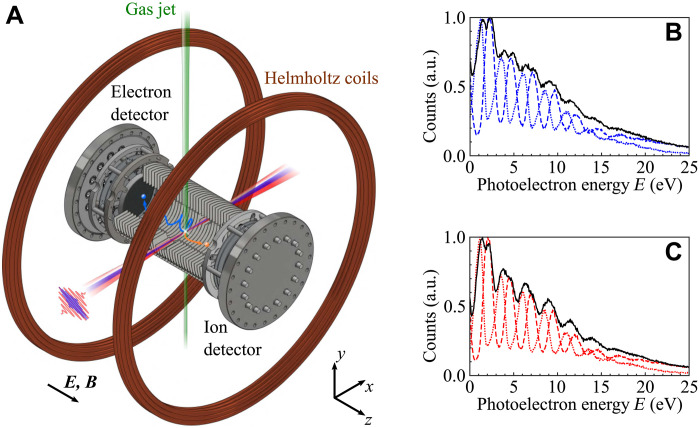
Coincidence spectroscopy in a molecular isotopic mixture. (**A**) Photoelectron-photoion coincidence spectrometer (ReMi) used in the experiment. (**B**) XUV-only photoelectron spectra measured in coincidence with the ionic fragments ${\mathrm{CH}}_{4}^{+}$ (blue dashed line) and ${\mathrm{CH}}_{3}^{+}$ (blue dotted line). (**C**) XUV-only photoelectron spectra measured in coincidence with the ionic fragments ${\mathrm{CD}}_{4}^{+}$ (red dashed line) and ${\mathrm{CD}}_{3}^{+}$ (red dotted line). The black lines are the normalized spectra obtained as the sum of those measured in coincidence with the ions ${\mathrm{CH}}_{4}^{+}$ and ${\mathrm{CH}}_{3}^{+}$ (B), and ${\mathrm{CD}}_{4}^{+}$ and ${\mathrm{CD}}_{3}^{+}$ (C). Adapted with permission from (*48*). a.u., arbitrary units.

We used a photoelectron-photoion coincidence spectrometer (reaction microscope, ReMi) (*27*–*30*) to disentangle the photoelectron spectra resulting from the photoionization of an equal mixture of ${\mathrm{CH}}_{4}$ and ${\mathrm{CD}}_{4}$ molecules (see Fig. 1A). The ion spectra measured using the XUV attosecond pulse train alone are dominated by the ionic fragments ${\mathrm{CH}}_{4}^{+}$ − ${\mathrm{CH}}_{3}^{+}$ and ${\mathrm{CD}}_{4}^{+}$ − ${\mathrm{CD}}_{3}^{+}$ (*31*, *32*). Figure 1 (B and C) shows the total photoelectron spectra (black lines), and those measured in coincidence with the ions ${\mathrm{CH}}_{4}^{+}$ and ${\mathrm{CH}}_{3}^{+}$ (blue dashed and dotted line, respectively) (Fig. 1B), and ${\mathrm{CD}}_{4}^{+}$ and ${\mathrm{CD}}_{3}^{+}$ (red dashed and dotted line, respectively) (Fig. 1C). The channel-resolved photoelectron spectra present a clear harmonic structure. The results are consistent with the measurements obtained by photoelectron-photoion coincidence spectroscopy in combination with monochromatic XUV radiation and are already available in the literature (see Experimental Information and fig. S1 in the Supplementary Materials) (*33*). The capability to measure in coincidence the photoelectrons and the corresponding ions is fundamental for characterizing the response of the two molecules under the same conditions, thus ruling out the effect of experimental instabilities, and to disentangle the contribution of the dissociating and non-dissociating ionization channels.

**Fig. 2.**
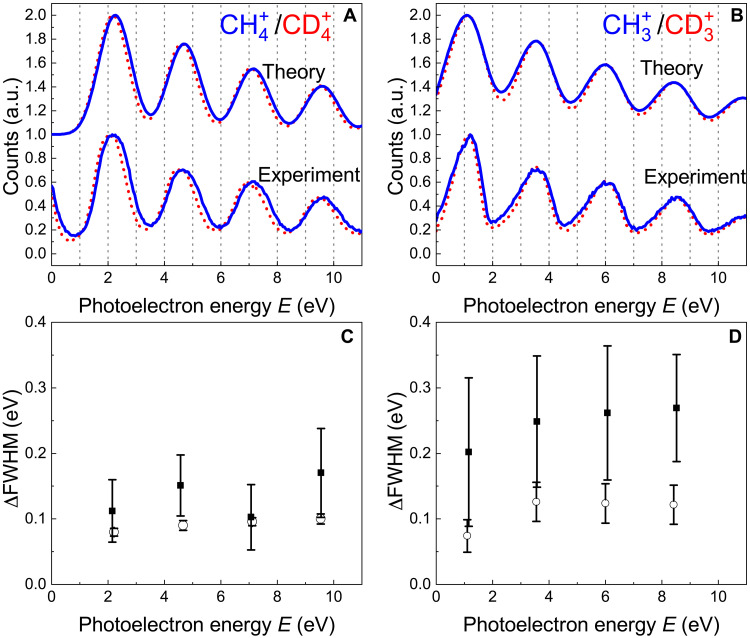
Isotopic dependence of channel-resolved XUV-only photoelectron spectra. Channel-resolved experimental (lower curves between 0 and 1) and theoretical (upper curves between 1 and 2) photoelectron spectra corresponding to the ions ${\mathrm{CH}}_{4}^{+}$ (blue solid line) and ${\mathrm{CD}}_{4}^{+}$ (red dotted line) (**A**) and to the ions ${\mathrm{CH}}_{3}^{+}$ (blue solid line) and ${\mathrm{CD}}_{3}^{+}$ (red dotted line) (**B**). The theoretical curves have been upshifted to one unity for the sake of clarity. Evolution of the experimental (full square) and simulated (open circles) differences ΔFWHM = ${\mathrm{FWHM}}_{{\mathrm{CH}}_{3,4}^{+}}-{\mathrm{FWHM}}_{{\mathrm{CD}}_{3,4}^{+}}$ of the FWHM width of the photoelectron peaks corresponding to the ions ${\mathrm{CH}}_{4}^{+}$ and ${\mathrm{CD}}_{4}^{+}$ (**C**) and to the ions ${\mathrm{CH}}_{3}^{+}$ and ${\mathrm{CD}}_{3}^{+}$ (**D**), respectively. The error bars were derived by the Gaussian fits of the corresponding experimental and simulated photoelectron peaks.

The width of a single photoelectron peak is broader than the bandwidth of the corresponding XUV harmonic, estimated at about 200 meV (full width at half maximum; FWHM). The broadening is due to the combination of the finite resolution of the photoelectron spectrometer (see Materials and Methods) and the spectral width of the vibronic absorption band of methane and deuteromethane (*33*). We observe that the width of the single photoelectron peak for the XUV-only photoionization process presents an isotopic dependence as shown in Fig. 2 (A and B). In particular, the FWHM of the single photoelectron peak measured in coincidence with the ionic channels ${\mathrm{CH}}_{4}^{+}$ and ${\mathrm{CH}}_{3}^{+}$ (lower curves drawn in blue solid lines in Fig. 2, A and B, respectively) is larger than the corresponding quantities measured in coincidence with the ions ${\mathrm{CD}}_{4}^{+}$ and ${\mathrm{CD}}_{3}^{+}$ (lower curves drawn in red dotted lines), respectively. This qualitative observation is quantified in Fig. 2 (C and D), which reports the difference of the FWHM (ΔFWHM = ${\mathrm{FWHM}}_{{\mathrm{CH}}_{3,4}^{+}}-{\mathrm{FWHM}}_{{\mathrm{CD}}_{3,4}^{+}}$; full squares for experimental data and open circles for numerical simulations) for the photoelectron peaks measured in coincidence with ions associated with the ${\mathrm{CH}}_{4}$ and ${\mathrm{CD}}_{4}$ molecules. As shown later, the broadening of the photoelectron peaks and the different widths can be explained by considering the autocorrelation function of the two isotopologues.

**Fig. 3.**
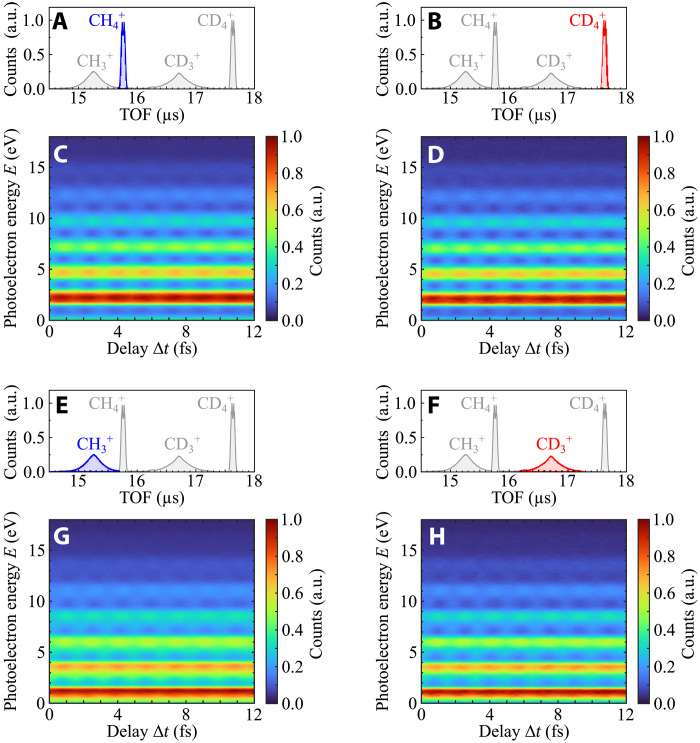
Channel-resolved RABBIT spectrograms measured in methane and deuteromethane. Ion time-of-flight (TOF) spectra highlighting the ions ${\mathrm{CH}}_{4}^{+}$ (**A**), ${\mathrm{CD}}_{4}^{+}$ (**B**), ${\mathrm{CH}}_{3}^{+}$ (**E**), and ${\mathrm{CD}}_{3}^{+}$ (**F**). RABBIT traces were obtained considering only photoelectrons measured in coincidence with the ion ${\mathrm{CH}}_{4}^{+}$ (**C**), ${\mathrm{CD}}_{4}^{+}$ (**D**), ${\mathrm{CH}}_{3}^{+}$ (**G**), and ${\mathrm{CD}}_{3}^{+}$ (**H**). Adapted with permission from (*48*).

When photoionization takes place in the presence of a synchronized IR field, additional photoelectron peaks (sidebands, SBs) appear between the main photoelectron lines, due to the interference between two photoionization pathways leading to the same final state. These involve the absorption of an XUV photon from consecutive harmonics and the absorption or emission of an additional IR photon by the outgoing photoelectron (*17*). The yield of the sidebands measured in coincidence with the ions ${\mathrm{CH}}_{4}^{+}$, ${\mathrm{CD}}_{4}^{+}$, ${\mathrm{CH}}_{3}^{+}$, and ${\mathrm{CD}}_{3}^{+}$ (see ion time of flight presented in Fig. 3, A, B, E, and F) clearly oscillates as a function of the relative delay Δ*t* between the attosecond pulse train and the IR field, as shown in Fig. 3 (C, D, G, and H, respectively). The amplitude offset (*A*_0ω_), oscillation amplitude (*A*_2ω_), and phase offset (φ) of these oscillations were obtained using the Fourier transform of the photoelectron spectra over an energy window of 300 meV around the sideband maxima and considering that the sideband signal is described by the relation

$$I_{\mathrm{SB}}(\text{Δ}t;E)=A_{0\omega }+A_{2\omega }\cos(2\omega \text{Δ}t-\varphi )$$ (1)

where ω indicates the frequency of the IR field driving the HHG process and *E* is the kinetic energy of the photoelectron (see also the “Experimental methods” section in Materials and Methods).

**Fig. 4.**
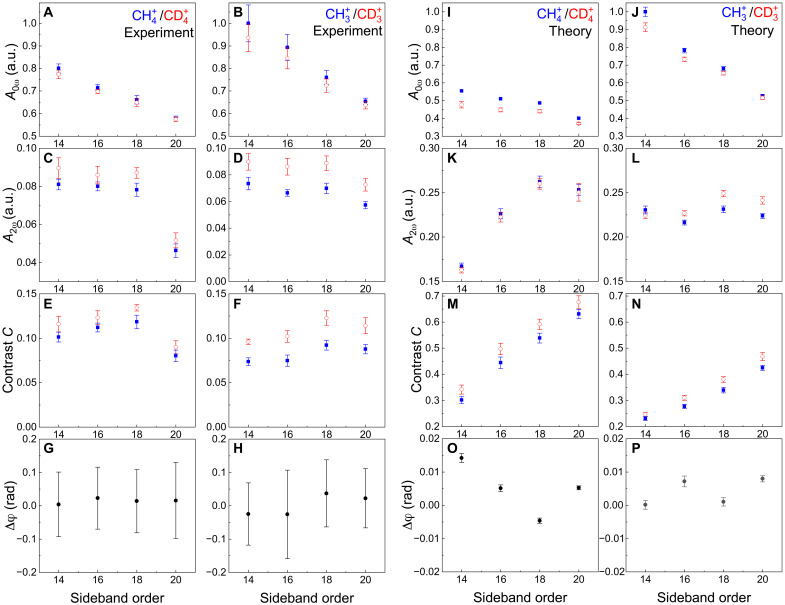
Experimental and simulated isotopic dependence of sideband oscillations in methane and deuteromethane. Comparison between the amplitude offset *A*_0ω_ (**A** and **B**), amplitude *A*_2ω_ (**C** and **D**), and contrast *C* = *A*_2ω_/*A*_0ω_ (**E** and **F**) between the experimental sidebands oscillations measured in coincidence with ${\mathrm{CH}}_{4}^{+}$ (blue full square) and ${\mathrm{CD}}_{4}^{+}$ (red open circle) (A), (C), and (E), and with ${\mathrm{CH}}_{3}^{+}$ (blue full square) and ${\mathrm{CD}}_{3}^{+}$ (red open circle) (B), (D), and (F) for different sideband orders. Phase difference Δφ (**G** and **H**) (black full circle) of the phase offset φ measured in coincidence with ${\mathrm{CH}}_{4}^{+}$ and ${\mathrm{CD}}_{4}^{+}$ (G), and with ${\mathrm{CH}}_{3}^{+}$ and ${\mathrm{CD}}_{3}^{+}$ (H) for different sideband orders. Panels (**I** to **P**) show the same quantities of panels (A) to (H) calculated from our theoretical simulations. Note the different *y* scales used for the experimental and theoretical data.

In Fig. 4, we present the comparison between the coefficients *A*_0ω_ (Fig. 4, A and B), *A*_2ω_ (Fig. 4, C and D), and the ratio *C* = *A*_2ω_/*A*_0ω_ (Fig. 4, E and F), which corresponds to the contrast of the sideband oscillations, for the ionic channels resulting from the photoionization of ${\mathrm{CH}}_{4}$ (blue full squares) and ${\mathrm{CD}}_{4}$ (red open circles). Moreover, we also show the channel-resolved difference of the phase of the sideband oscillations Δφ for the channels ${\mathrm{CH}}_{4}^{+}$ − ${\mathrm{CD}}_{4}^{+}$ (Fig. 4G) and ${\mathrm{CH}}_{3}^{+}$ − ${\mathrm{CD}}_{3}^{+}$ (Fig. 4H). A detailed description of the analysis followed to isolate the different terms is presented in the Supplementary Materials.

The amplitude offsets *A*_0ω_ are slightly larger for the ${\mathrm{CH}}_{4}^{+}$ channel than for ${\mathrm{CD}}_{4}^{+}$ (Fig. 4A), as well as for ${\mathrm{CH}}_{3}^{+}$ with respect to ${\mathrm{CD}}_{3}^{+}$ (Fig. 4B). The amplitude of the oscillations *A*_2ω_ presents an opposite behavior, as shown in Fig. 4 (C and D), with a clear isotopic difference between ${\mathrm{CH}}_{3}^{+}$ and ${\mathrm{CD}}_{3}^{+}$ (Fig. 4D). As a result, the contrast *C* of the sideband oscillations turns out to be larger in the fragments originating from the ${\mathrm{CD}}_{4}$ molecule (Fig. 4, E and F). The differences of the phases φ for the pairs ${\mathrm{CH}}_{4}^{+}$ − ${\mathrm{CD}}_{4}^{+}$ and ${\mathrm{CH}}_{3}^{+}$ − ${\mathrm{CD}}_{3}^{+}$ do not present a substantial variation (Fig. 4, G and H). The experimental data indicate the presence of isotopic effects in the two-color photoionization process that manifest themselves in the amplitude and contrast of the sideband oscillations, and not in the phase of the oscillations. The effect on the contrast suggests that the coherence properties of the two-color photoionization (*34*) process are affected by the isotopic substitution. We have verified that the evolution of the parameters *A*_0ω_, *A*_2ω_, and φ as a function of the photoelectron kinetic energy and the presence of isotopic effects do not notably depend on the integration width, thus indicating the robustness of the analysis.

The RABBIT traces were simulated using perturbation theory, including up to two-photon effects to take into account the exchange of two photons with the XUV-IR field (see Theoretical Model, figs. S2 to S11, and tables S1 and S2 in the Supplementary Materials) (*35*). One- and two-photon electronic matrix elements were calculated using the stationary multiphoton molecular R-matrix approach (*36*, *37*). The effect of nuclear dynamics in the XUV-only and in the XUV-IR photoionization processes was modeled using the vibronic autocorrelation function *A*(τ) (*38*), where τ indicates the time elapsed between the two interfering pathways leading to the same sideband. The autocorrelation function expresses the overlap between the time-dependent nuclear wave packet χ(*q*, τ) after the time delay τ and the initial wave packet χ(*q*, 0) created in the cation (*q* indicates the nuclear coordinate). A closely related approach was already adopted to describe nuclear-motion effects in HHG (*12*, *16*, *39*, *40*) and attosecond electron-hole migration (*8*, *41*, *42*). The modulation depth *B*(*p*) of the sideband as a function of the final electron momentum *p* is proportional to the product of the electronic matrix element *M*(*p*) and the Fourier transform of the nuclear autocorrelation *N*(ɛ*_p_*)

$$B(p)\propto M(p)N(\varepsilon_{p})$$ (2)

where ɛ*_p_* is the vibrational energy and *M*(*p*) is averaged over the ground state (zero-point energy) vibrational function (see equations 7 and 8 in the Supplementary Materials). The photoelectron kinetic energy *E* = *p*^2^/2*m_e_* (with *m_e_* being the electron mass) is related to the vibrational energy ɛ*_p_* by the relation: *E* = −ɛ*_p_* + *nℏ*ω − *I_p_*, where *n* and *I_p_* indicate the harmonic or sideband order and the vertical ionization potential, respectively. This expression indicates that the energy absorbed by the molecule from the one- or two-color field is redistributed between the kinetic energy of the photoelectron *E* and the vibrational energy of the cation ɛ*_p_*.

**Fig. 5.**
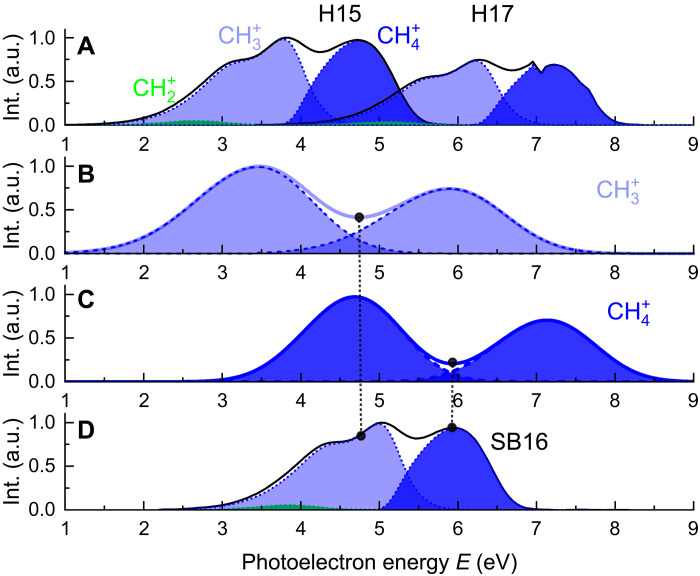
Simulated XUV-only and two-color photoelectron spectra. (**A**) Main photoelectron lines (black solid line) determined by the absorption of a single XUV photon of the harmonics H15 and H17 in ${\mathrm{CH}}_{4}$. The shaded areas indicate the photoelectron spectra associated with the ionic channels ${\mathrm{CH}}_{2}^{+}$ (green), ${\mathrm{CH}}_{3}^{+}$ (light blue), and ${\mathrm{CH}}_{4}^{+}$ (blue). (**B** and **C**) Simulated XUV-only photoelectron spectra (full solid line) considering only the contributions of the photoelectrons associated with ${\mathrm{CH}}_{3}^{+}$ (B) and ${\mathrm{CH}}_{4}^{+}$ (C) and the harmonics H15 and H17. The dashed lines and shaded areas indicate the simulated spectra obtained considering a single harmonic and the convolution with the response function of the ReMi photoelectron spectrometer. (**D**) Profile of the photoelectron sideband SB16 (black line) and its decomposition in the contributions associated with the ionic channels ${\mathrm{CH}}_{2}^{+}$ (green), ${\mathrm{CH}}_{3}^{+}$ (light blue), and ${\mathrm{CH}}_{4}^{+}$ (blue). The vertical black dashed lines indicate the positions of the minima of the XUV-only photoelectron spectra associated with the ions ${\mathrm{CH}}_{3}^{+}$(left) and ${\mathrm{CH}}_{4}^{+}$ (right).

The simulated photoelectron spectra due to the absorption of a single XUV photon from the harmonic order *n* = 15 and *n* = 17 are presented in Fig. 5A (black lines). The overall spectral shape is dominated by the shape of the function *N*(ɛ*_p_*) and matches the photoelectron spectra measured with monochromatic XUV radiation well (*33*) (see fig. S1A in the Supplementary Materials).

The overall photoelectron spectra can be decomposed into three contributions corresponding to the dissociative (${\mathrm{CH}}_{2}^{+}$ and ${\mathrm{CH}}_{3}^{+}$) and non-dissociative (${\mathrm{CH}}_{4}^{+}$) channels (*43*), respectively. We used filters based on the measured branching ratios (*33*) to isolate these three contributions (see fig. S1B), thus reproducing the ion-resolved photoelectron detection of the experiment. The result is represented by the shaded areas shown in Fig. 5A for the channel ${\mathrm{CH}}_{2}^{+}$ (green area), ${\mathrm{CH}}_{3}^{+}$ (light blue area), and ${\mathrm{CH}}_{4}^{+}$ (blue area). The XUV-only spectrum is constructed as the sum of the contributions of the different harmonics (H15 and H17 in Fig. 5), convoluted with the response function of the photoelectron spectrometer, as shown for the spectrum associated with the ions ${\mathrm{CH}}_{3}^{+}$ (Fig. 5B) and ${\mathrm{CH}}_{4}^{+}$ (Fig. 5C), respectively. A similar procedure considering the autocorrelation function for ${\mathrm{CD}}_{4}$ was used for simulating the XUV-only spectra associated with the ions ${\mathrm{CD}}_{3}^{+}$ and ${\mathrm{CD}}_{4}^{+}$.

The simulated XUV-only spectra reproduce the main features of the experimental photoelectron spectra (see upper curves in Fig. 2, A and B). The difference of the spectral widths of the simulated peaks (open circles) matches qualitatively those of the experimental photoelectron peaks (full square) measured in coincidence with the ions ${\mathrm{CH}}_{4}^{+}$ − ${\mathrm{CD}}_{4}^{+}$ and ${\mathrm{CH}}_{3}^{+}$ − ${\mathrm{CD}}_{3}^{+}$, as shown by the comparison in Fig. 2 (C and D, respectively). In particular, the larger width of the experimental photoelectron peaks associated with the ionic channels originating from ${\mathrm{CH}}_{4}$ with respect to those derived from the photoionization of ${\mathrm{CD}}_{4}$ is qualitatively reproduced in the simulations.

The good agreement between the channel-resolved experimental data measured in the XUV-only case and the simulated spectra allows one to extend the approach to the two-color photoionization process for both isotopologues, including also the contribution of the sideband photoelectron peaks. Figure 5D presents the simulated spectrum for the sideband SB16, together with the filtered contributions corresponding to the ionic channels ${\mathrm{CH}}_{2}^{+}$ (green area), ${\mathrm{CH}}_{3}^{+}$ (light blue area), and ${\mathrm{CH}}_{4}^{+}$ (blue area). As expected, the maxima of the sidebands associated with the ${\mathrm{CH}}_{3}^{+}$ (light blue) and ${\mathrm{CH}}_{4}^{+}$ (blue) channels correspond to the minima between consecutive harmonics of the corresponding XUV spectrum, as indicated by the vertical black dashed lines in Fig. 5 (B to D). We constructed the complete RABBIT traces generated by the two-color process as the incoherent sum of the high (low) energy contributions of the main photoelectron lines and of the sideband peaks for the ionic channels ${\mathrm{CH}}_{4}^{+}$ (${\mathrm{CH}}_{3}^{+}$). The incoherent sum is justified by the observation that the one- and two-photon pathways leading to the same final photoelectron energy will access different vibrational states of the ion, therefore suppressing the interference between the two paths. The validity of this approach is supported by the good agreement between the delay (Δ*t*) integrated experimental and simulated RABBIT traces (see fig. S12).

The simulated RABBIT spectrograms (reported in fig. S13) were analyzed to extract the coefficients *A*_0ω_, *A*_2ω_, and φ. The results are presented in Fig. 4 (I to P) and are in close qualitative agreement with the experimental findings. The model reproduces the differences in *A*_0ω_ between the ionic channels observed in the experiment (see Fig. 4, I and J). In particular, the amplitude offsets *A*_0ω_ of the sidebands associated with the ionic fragments ${\mathrm{CH}}_{4}^{+}$ (Fig. 4I) and ${\mathrm{CH}}_{3}^{+}$ (Fig. 4J) are larger than the corresponding quantities in ${\mathrm{CD}}_{4}^{+}$ and ${\mathrm{CD}}_{3}^{+}$. Furthermore, the model reproduces the trends in the amplitudes *A*_2ω_, with a negligible difference for the pair ${\mathrm{CH}}_{4}^{+}$ − ${\mathrm{CD}}_{4}^{+}$ (Fig. 4K) and a substantial one for the ionic fragments ${\mathrm{CH}}_{3}^{+}$ − ${\mathrm{CD}}_{3}^{+}$ (Fig. 4L). These observations are in qualitative agreement with the experimental results shown in Fig. 4 (C and D). As in the experiment, the final result is an increased contrast *C* of the oscillations for the sidebands originating from the ${\mathrm{CD}}_{4}$ molecule (Fig. 4, M and N), which is particularly pronounced in the comparison ${\mathrm{CD}}_{3}^{+}$ − ${\mathrm{CH}}_{3}^{+}$ (Fig. 4N).

However, the experimental data and simulations also present notable differences. The large deviation of the amplitude *A*_2ω_ for SB20 for ${\mathrm{CH}}_{4}^{+}$ from the other experimental points (see Fig. 4C) might be ascribed to the reduced spectrometer resolution at high photoelectron kinetic energies and to averaging over the interaction volume. Similarly, the reduced value of the amplitude for SB14 observed in the theoretical simulations (see Fig. 4K) might indicate a limitation of the model in describing the two-color photoionization process close to the ionization threshold of the molecule. Although isotopic effects can be observed when comparing the contrast for different isotopologues, these discrepancies could be responsible for the different evolution of the contrast *C* as a function of the sideband order between theory and experiment for the non-dissociative channel.

In general, the amplitude of the oscillations at frequency 2ω and the contrast estimated from the numerical model are higher than those measured in the experiment. We attribute these differences to the effect of averaging over the interaction volume and small distortions in the electric and magnetic fields used in the photoelectron spectrometer, which might lead to partial smearing out of the sideband oscillations in the experimental data. Last, the model also reproduces the absence of significant variation in the phase difference between homolog isotopic species (${\mathrm{CH}}_{4}^{+}$ − ${\mathrm{CD}}_{4}^{+}$ and ${\mathrm{CH}}_{3}^{+}$ − ${\mathrm{CD}}_{3}^{+}$) (Fig. 4, O and P).

## DISCUSSION

**Fig. 6.**
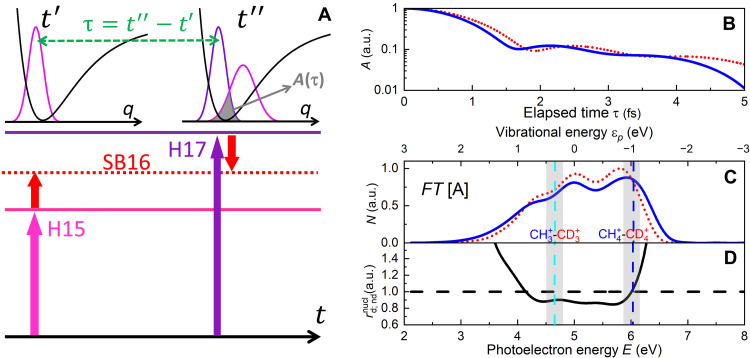
Effect of nuclear dynamics on RABBIT signal and role of the nuclear autocorrelation function. (**A**) Schematic view of the effect of nuclear dynamics on the RABBIT signal in molecules. The times *t*′ and *t*″ indicate the instants at which the transitions from the ground state to the cation + continuum state occur. The potential energy surface of the cation along the nuclear coordinate *q* is represented schematically by black curves. The magenta curves represent schematically the nuclear wave packet launched in the molecular cation by the two-color path at time *t*′ and after the propagation for time τ = *t*″ − *t*′. The violet curve represents schematically the nuclear wave packet launched in the molecular cation by the second two-color path at time *t*″. The overlap between the two nuclear wave packets at *t*″ is represented by the gray area. (**B**) Nuclear autocorrelation function *A* for ${\mathrm{CH}}_{4}$ (blue full line) and ${\mathrm{CD}}_{4}$ (red dotted line). (**C**) Fourier transform of the autocorrelation function for ${\mathrm{CH}}_{4}$ (blue full line) and ${\mathrm{CD}}_{4}$ (red dotted line) shifted in the photoelectron kinetic energy region of the SB16. (**D**) Ratio of the Fourier transform of the autocorrelation functions of ${\mathrm{CH}}_{4}$ and ${\mathrm{CD}}_{4}$. The horizontal dashed line indicates the value $r_{d,\mathrm{nd}}^{\mathrm{nuc}}=1$. The vertical dashed lines correspond to the energy position of the center of the sideband photoelectrons associated with the ionic channels ${\mathrm{CH}}_{3}^{+}$ − ${\mathrm{CD}}_{3}^{+}$ (light blue) and ${\mathrm{CH}}_{4}^{+}$ − ${\mathrm{CD}}_{4}^{+}$ (blue). The gray shaded regions indicate the energy interval width of 300 meV corresponding to the sideband photoelectrons associated with the ionic channels ${\mathrm{CH}}_{3}^{+}$ − ${\mathrm{CD}}_{3}^{+}$ (left area) and ${\mathrm{CH}}_{4}^{+}$ − ${\mathrm{CD}}_{4}^{+}$ (right area) (see also vertical lines in Fig. 5, C and D).

The interference term of the two-color RABBIT signal corresponding to a sideband (for example, SB16 in Fig. 6A) is contributed by two pathways characterized by the absorption of one XUV (H15) and one IR photon, and by one photon of the consecutive harmonic (H17) and the emission of one IR photon. Both paths contribute to the formation of the same photoelectron sideband in the continuum. In our theoretical model, pairs of pathways occurring at different instants (indicated as *t*′ and *t*″) spaced by a time τ interfere (*35*). The time interval τ affects the interference between the two pathways, depending on the dynamics of the nuclear wave packet of the cation. The first path launches a nuclear wave packet in the cation (schematically represented in magenta) that evolves along its potential energy surface (schematically shown in black) for the time interval τ, when the second pathway takes place, projecting the associated wave packet in the cation (violet curve). For very short times, the dominant dynamics occurring in the cations are predicted to be the *D*_2*d*_ distortion, toward the minima of the diabatic electronic surfaces (*16*, *39*, *40*). The interference between the two paths (and therefore the contrast of the oscillation of the sideband intensity) depends on the overlap between the wave packets launched with the relative delay τ. This overlap is shown as a gray shaded area in Fig. 6A and is described by the autocorrelation function *A*(τ) presented in Fig. 6B for the cations of ${\mathrm{CH}}_{4}$ (blue solid line) and ${\mathrm{CD}}_{4}$ (red dotted line). Both functions present an ultrafast decay in the very first few femtoseconds. Moreover, due to the lighter mass of the hydrogen atoms with respect to the deuterium ones, the autocorrelation function for the cations of ${\mathrm{CH}}_{4}$ presents a faster decay with respect to that of ${\mathrm{CD}}_{4}$. This condition determines a reduced overlap between the two wave packets in the cation of ${\mathrm{CH}}_{4}$ with respect to the deuterated isotopologues, resulting in a reduced contrast for the sidebands measured in coincidence with the ionic channels resulting from ${\mathrm{CH}}_{4}$. This simple physical interpretation, derived from our theoretical description of the molecular RABBIT [see the Supplementary Materials and (*35*)], is supported by the experimental results presented in Fig. 4.

The interference between the pathways labeled by all possible launching times *t*′ and *t*″ can be quantified by considering the Fourier transform *N*(ɛ*_p_*) of the nuclear autocorrelation function *A* in the spectral domain presented in Fig. 6C (hereafter, we will use subscripts H and D to indicate quantities related to the isotopologues ${\mathrm{CH}}_{4}$ and ${\mathrm{CD}}_{4}$, respectively).

In the energy domain, the difference between the decay times determines a different broadening of their Fourier transforms, resulting in the function *N*_H_ (blue solid line) being broader than *N*_D_ (red dotted line) both at low and at high energies, as shown in Fig. 6C.

The shape of the functions *N*_H,D_ reproduces well the photoelectron spectra generated by each harmonic of the XUV spectrum (see fig. S1A). Moreover, the single photoelectron peak can be decomposed in a high-energy part associated with the ionic channels ${\mathrm{CH}}_{4}^{+}$ − ${\mathrm{CD}}_{4}^{+}$ and low energy contribution associated (mainly) with ${\mathrm{CH}}_{3}^{+}$ − ${\mathrm{CD}}_{3}^{+}$ (see Fig. 5). The combination of these observations indicates that the larger broadening of the photoelectron peaks measured for the XUV-only case in coincidence with the ionic channel ${\mathrm{CH}}_{4}^{+}$ with respect to ${\mathrm{CD}}_{4}^{+}$ is due to the larger width of the function *N*_H_ at high energies (see Fig. 2, A and C). Similarly, the larger broadening of the photoelectron peaks measured in coincidence with the ionic channel ${\mathrm{CH}}_{3}^{+}$ is due to the differences of the functions *N*_H_ and *N*_D_ for the two isotopologues at low energies (see Fig. 2, B and D).

In the two-color photoionization measurements, the differences observed in the amplitudes *A*_0ω_ can be also explained by the different spectral widths of the functions *N*_H_ and *N*_D_. The larger *A*_0ω_ component in ${\mathrm{CH}}_{4}^{+}$ and ${\mathrm{CH}}_{3}^{+}$ with respect to their deuterated counterparts can be ascribed to the larger width of the XUV-only spectra, which determines a larger contribution of the photoelectron released by the absorption of a single XUV photon to the energy intervals between consecutive harmonics, where the two-photon (XUV-IR) sideband signal is located (see Fig. 5D).

**Fig. 7.**
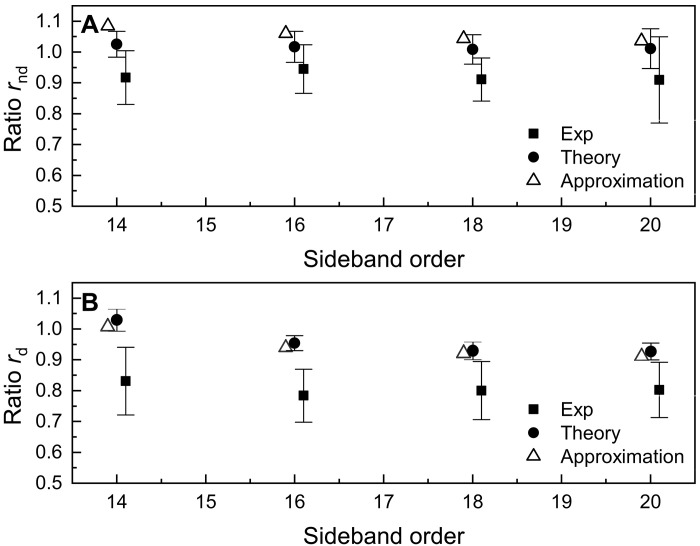
Ratios of the sideband oscillations for dissociating and non-dissociating channels. Ratio *r*_nd_ (**A**) and *r*_d_ (**B**) for different sideband orders of the experimental (square) and the theoretical data (circle). The error bars were determined by error propagation of the error bars of the quantities *A*_2ω_ shown in Fig. 4. The triangles represent the value of the ratios obtained *r*_d,nd_ as the product of the electronic and nuclear contributions (see Eq. 3).

For the interpretation of the evolution in the *A*_2ω_ components, we observe that the difference between the non-dissociating channels ${\mathrm{CH}}_{4}^{+}$ − ${\mathrm{CD}}_{4}^{+}$ is notably smaller than for the dissociating channels ${\mathrm{CH}}_{3}^{+}$ − ${\mathrm{CD}}_{3}^{+}$, both in the experiment (Fig. 4, C and D) and in the simulations (Fig. 4, K and L). We can quantify this difference by introducing the ratio between the amplitude of the sideband oscillations for the dissociating [*r*_d_ = *A*_2ω_(${\mathrm{CH}}_{3}^{+}$)/*A*_2ω_(${\mathrm{CD}}_{3}^{+}$)] and non-dissociating [*r*_nd_ = *A*_2ω_(${\mathrm{CH}}_{4}^{+}$)/*A*_2ω_(${\mathrm{CD}}_{4}^{+}$)] channels. The ratio *r*_nd_ is very close to one and larger than *r*_d_ both in the experiment and simulations, as shown in Fig. 7 (A and B) (see also table S3).

In our numerical model, the amplitude of the oscillations of the sideband intensities *A*_2ω_ is proportional to the modulus of the Fourier transform of the modulation depth *B*(*p*) at the frequency 2ω (see Eq. 2). This can be expressed as the product of the modulus of an electronic term (∣*M*_H,D_∣) (shown in fig. S14A) and a nuclear contribution given by the function *N*_H,D_. As a result, the ratios *r*_d_ and *r*_nd_ can be expressed as the product of a ratio for the electronic contribution ($r_{d,\mathrm{nd}}^{\mathrm{el}}$) and one for the nuclear part ($r_{d,\mathrm{nd}}^{\mathrm{nucl}}$):

$$r_{d,\mathrm{nd}}=\frac{|F{\left[B_{H}\right]}_{2\omega }|}{|F{\left[B_{D}\right]}_{2\omega }|}=\underset{r_{d,\mathrm{nd}}^{\mathrm{el}}}{\underbrace{\frac{|M_{H}|}{|M_{D}|}}}\underset{r_{d,\mathrm{nd}}^{\mathrm{nucl}}}{\underbrace{\frac{N_{H}}{N_{D}}}}$$ (3)

In the model, the ratios of the electronic terms depends only weakly on the dissociating or non-dissociating channel, through its energy dependence. For example, they assume the values $r_{d}^{\mathrm{el}}=1.052$ and $r_{\mathrm{nd}}^{\mathrm{el}}=1.042$ at the center of the sideband SB16 for the dissociating and non-dissociating channels, respectively (see fig. S14B). The values for the other sidebands are reported in table S3, indicating that the electronic part of the amplitude of the sideband oscillation in ${\mathrm{CH}}_{4}$ is larger than in ${\mathrm{CD}}_{4}$. This ratio is modified by the nuclear contribution as shown in Fig. 6C; at the center of the region corresponding to the sidebands measured in coincidence with the ion ${\mathrm{CH}}_{3}^{+}$ − ${\mathrm{CD}}_{3}^{+}$ (indicated by the light blue vertical dashed line), the function *N*_H_ (blue solid line) is smaller than *N*_D_ (red dotted line), leading to a ratio of the nuclear contribution $r_{d}^{\mathrm{nucl}}$ lower than one, which compensates the ratio of the electronic part $r_{d}^{\mathrm{el}}$. For the non-dissociating channel ${\mathrm{CH}}_{4}^{+}$ − ${\mathrm{CD}}_{4}^{+}$ (vertical blue dashed line), the ratio of the two curves is close to one.

We note that both in the experiment and in the simulations, the sideband photoelectrons are evaluated over an energy region (gray shaded areas in Fig. 6, B and C) of about 300 meV around the sideband maxima. In the case of the dissociation channel (${\mathrm{CH}}_{3}^{+}$ and ${\mathrm{CD}}_{3}^{+}$) (gray shaded area on the left-hand side), the function *N*_H_ remains smaller than *N*_D_ over the entire interval, while for the non-dissociating channel (${\mathrm{CH}}_{4}^{+}$ and ${\mathrm{CD}}_{4}^{+}$; gray shaded area on the right-hand side), the two curves cross. The nuclear contribution $r_{d,\mathrm{nd}}^{\mathrm{nucl}}$ can be approximated as the ratio of the integrals of the curves *N*_H,D_ over the energy intervals corresponding to the sidebands, returning the values $r_{d}^{\mathrm{nucl}}=0.894$ and $r_{\mathrm{nd}}^{\mathrm{nucl}}=1.017$ (the results are summarized in table S3). The factorization of the ratio *r*_d,nd_ in an electronic and nuclear contribution and its approximate estimation (open triangles in Fig. 7) is in good quantitative agreement with the results from the theoretical simulations (full circles) and in fair agreement with the experimental results (full squares).

In the theoretical model, the phase difference Δφ between the phases of the sideband oscillations of different isotopic channels originates predominantly from the energy dependence of the electronic part of the matrix dipole moment, as the nuclear motion introduces only a minor correction. This property is a direct consequence of the negligible action of the IR laser field on the cation, which leads to time-reversible dynamics. The nuclear autocorrelation function is then Hermitian with respect to time reversal and its Fourier transform is guaranteed to be real and positive semidefinite (see equations 9 and 10 in the Supplementary Materials). As a result, the nuclear motion does not add any phase to the photoionization matrix element. The main isotope-dependent contribution to the phase difference is due to the different extent of the zero-point initial wave functions in the two isotopologues, but this term (≈0.01 rad; see Fig. 4, O and P) turns out to be well below the typical experimental error bars (≈0.1 rad; see Fig. 4, G and H).

Because the fundamental reason for the vanishing contribution to the phase of the nuclear dynamics is its time reversibility, one can envision experimental conditions breaking the time symmetry by preparing a nuclear wave packet in the neutral species. This can be realized, for example, by impulsive Raman excitation using a preceding pump pulse. Another possibility for observing an isotope-dependent RABBIT phase is to enhance the coordinate dependence of the electronic matrix elements, by choosing a sideband near a zero crossing of the corresponding electronic matrix element.

In conclusion, we have shown that nuclear dynamics notably affects the amplitude of the two-color photoionization signal in attosecond photoelectron interferometry in molecules. The effects can be interpreted in the spectral domain considering the Fourier transform of the autocorrelation function. The implementation of two isotopologues allows one to highlight the effect of nuclear dynamics by exploiting the different decay times determined by the isotopic substitution. While the investigation of nuclear dynamics in HHG using different isotopologues only gives access to cation dynamics on the time scale of the laser-cycle period, the RABBIT technique is potentially sensitive to longer time scales, up to the XUV or IR pulse duration. Moreover, while HHG-based investigations can only probe the dynamics of the ground state of the cation, or, at most, one of its low-lying (within a few electron volts) excited states (due to the exponential sensitivity of the strong-field ionization to the ionization potential), RABBIT can access short- and intermediate-time dynamics of any cationic state, as long as the photoelectron signal from different electronic states can be experimentally separated, either through the fragmentation-channel detection or photoelectron-energy selection. The short duration of the autocorrelation in the time domain (only a few femtoseconds) introduces a finite coherence time for the interaction of the attosecond pulse trains with the molecule. The coherence of the correlated electronic-nuclear wave packets is expected to play a major role in several molecular systems characterized by ultrafast dynamics after the photoionization event (*8*) and for the advancement of attosecond metrology (*34*).

## MATERIALS AND METHODS

### Experimental methods

Trains of attosecond pulses with photon energies up to 50 eV were generated in krypton using 20-fs driving IR pulses centered at λ = 1012 nm at a 50-kHz repetition rate. The intensity of the field driving the HHG process was estimated in *I* = 10^14^ W/cm^2^. The temporal delay between the XUV radiation and the IR field was changed in a collinear geometry using a pair of drilled plates (*44*, *45*). The two-color field was focused on the interaction point of the ReMi using a toroidal mirror operating in one-to-one imaging at an incidence angle of 84°. The gas target was composed of an equal mixture of ${\mathrm{CH}}_{4}$ and ${\mathrm{CD}}_{4}$ molecules. The typical count rate in the measurements was 5 to 6 kHz and data were acquired for 96 hours. The FWHM of the response function of the ReMi photoelectron spectrometer was estimated at ≈1000 meV. The data discussed in the manuscript were integrated over all emission directions and all orientations of the molecules. The RABBIT spectrograms were analyzed by Fourier transforming the signal at each energy and extracting the parameters *A*_0ω_, *A*_2ω_, and φ. The values in the manuscript correspond to the mean value of the respective quantities over an energy window of 300 meV. The error bars are given by the SD of the respective quantities over the same energy window.

### Theoretical methods

One- and two-photon photoionization matrix elements were calculated using the development version of UKRmol+ code (*46*) and aug-cc-pVTZ atomic basis set was used for the bound states. The continuum basis consisted of a mixed set of Gaussians reaching to the distance of 7.5 atomic units (au), followed by 10 B-splines spanning the remaining distance to the boundary of the R-matrix inner region at *a* = 15 au. The calculated energy of the ground neutral state was adjusted so that it was exactly 14.4 eV below the calculated energy of the ground ionic state. Bound molecular orbitals were calculated using the three-state state-averaged complete active space self-consistent field method with restricted open-shell Hartree-Fock reference for ${\mathrm{CH}}_{4}^{+}$ in PSI4 v.1.5 (*47*). The lowest three states were equally weighted. Carbon 1-s orbitals were frozen in the close-coupling calculations. The next 11 lowest molecular orbitals were used as active, both for the bound-state complete active space calculation and to build the square-integrable part of the close-coupling expansion. The continuum part of the expansion used 100 cationic states. Equilibrium geometries and force-fields for the neutral species were calculated at the MP2(fc)/aug-cc-pVTZ level. The quadratic vibronic Hamiltonian for the cation was obtained by diabatizing MR-CIS/CASSCF(7,4) energies of the ^2^*T*_2_ levels in the vicinity of the neutral equilibrium structure. The autocorrelation functions were evaluated for 300 au of time (≈7.26 fs), correponding to maximum spectral resolution of ≈0.3 eV. Zero-point corrections to the electronic matrix were evaluated using finite displacements along normal modes, with seven distorted structures required for each isotopomer. Orientational averaging was performed using order-17 Lebedev grids. Further details of the numerical parameters and procedures are given in the Supplementary Materials.

## Supplementary Materials

### This PDF file includes:

Experimental Information Theoretical Methods Figs. S1 to S14 Tables S1 to S3 References
