# Supplementary material for: Influence of nuclear dynamics on molecular attosecond photoelectron interferometry
Source: Sci Adv. 2023 Sep 30;9(35):eadh7747. doi: 10.1126/sciadv.adh7747 (PMC10468127; doi:10.1126/sciadv.adh7747)
Supplement: Supplementary file 1 — Experimental Information Theoretical Methods Figs. S1 to S14 Tables S1 to S3 References [file sciadv.adh7747_sm.pdf]

Science Advances
AAAS

Supplementary Materials for

Influence of nuclear dynamics on molecular attosecond photoelectron interferometry

Dominik Ertel *et al.*

Corresponding author: Giuseppe Sansone, giuseppe.sansone@physik.uni-freiburg.de

Sci. Adv. **9**, eadh7747 (2023)
DOI: 10.1126/sciadv.adh7747

This PDF file includes:

Experimental Information
Theoretical Methods
Figs. S1 to S14
Tables S1 to S3
References

1 Experimental Information

Methane CH_4 and deuteromethane CD_4 present a tetrahedral structure and belong to the T_d symmetry point group. The ground state electronic configuration is $(1a_1)^2(2a_1)^2(1t_2)^6$. Ionization of one $(1t_2)^{-1}$ electron results in the formation of the cationic $\tilde{X}^2T_2$ ground state. Ionization of one $2a_1$ electron leads to the A excited state. The adiabatic ionization energies for the ground and first excited cationic states are 12.61 eV and 22.39 eV, respectively (49). The XUV spectrum used in the experiment extends to energies up to ≈50 eV giving access to ionization from both states. Upon photoionization from the outermost shell, the ground state of the cation CH_4^+ (CD_4^+) $\tilde{X}^2T_2$ undergoes a dynamical rearrangement due to the Jahn-Teller effect, which induces a coupling between the three degenerate electronic ground states of the cation and the degenerate vibrational excitation with symmetry t and e. The vibronic coupling is responsible for the large width of the first vibronic band (33). This band is associated to the ionic channels CH_4^+, CH_3^+, and CH_2^+ (50, 51).

Photoionization resulting in the excited cationic state A is accompanied with the formation of the ionic channels CH_3^+, CH_2^+, and CH^+ (33). Indeed, the cation CH_4^+ is unstable for ionization energies above 14.4 eV. The formation of the cation CH_3^+ in the A state accounts for at most 0.4% of the total cross section (33), leading to the conclusion that the photoelectron spectra measured in coincidence with the two ionic channels CH_4^+ and CH_3^+ results essentially only from the cationic ground state.

The photoelectron spectra measured with monochromatic XUV radiation (data taken and adapted from ref. (33)) is presented in Fig. S1a) and well matches the shape of the autocorrelation function $N(E)$ (the energy axis was shifted to fit the central photon energy corresponding to the harmonic H15). Figure S1b shows the experimental branching ratios in the dissociative (CH_2^+ and CH_3^+) and non-dissociative (CH_4^+) channels (33, 43), together with the fitting

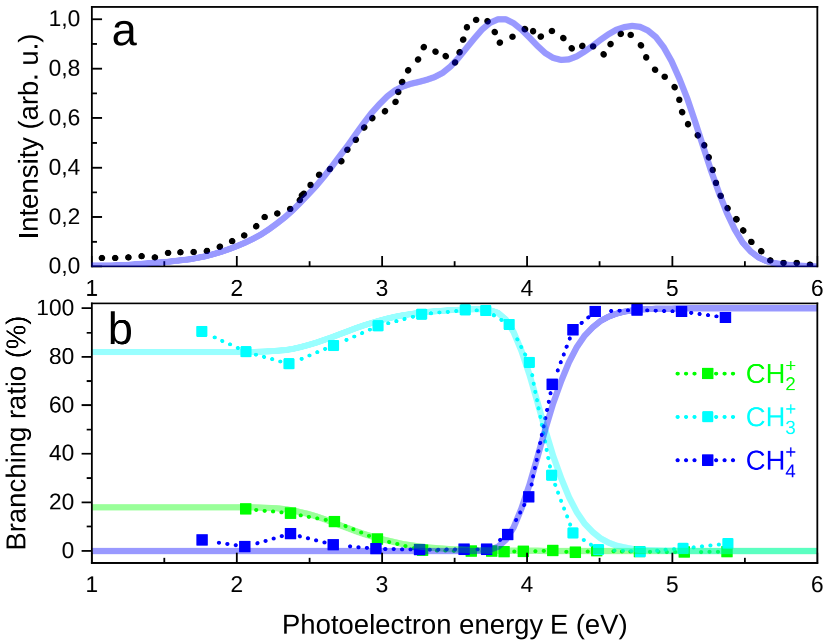

Figure S1: Experimental and simulated photoelectron spectrum and branching ratios in CH_4. (a) Experimental (black dotted line) and simulated (blue line) photoelectron spectrum generated by the photon energy of the harmonic H15 ($\hbar\omega = 18.25$ eV) in CH_4. (b) Experimental (square symbols and dotted lines) and adapted (full solid lines) branching ratios for the ionic fragments CH_2^+ (green), CH_3^+ (light blue), and CH_4^+ (blue). The experimental data taken from ref. (33) were adapted considering the photon energy difference between the HeI line and the harmonic H15 used in this work.

functions used to simulate the single and two-color photoelectron spectra.

2 Theoretical methods

The general-case expressions for modelling molecular RABBIT signal are given elsewhere (35). Here, we recapitulate the expressions needed to evaluate the contributions discussed in the manuscript. The signal at final photoelectron momentum p is given by a sum of three contributions:

$$I(p) = I_{1-,1-}(p) + I_{2+,2+}(p) + B(p), \tag{1}$$

$$B(p) = 2\Re[I_{1-,2+}(p)]. \tag{2}$$

The first contribution is the photoelectron signal due to the simultaneous absorption of a photon with frequency $\Omega_1 = (M-1)\omega$ and one with frequency ω. The second term describes the absorption of a photon with frequency $\Omega_2 = (M+1)\omega$ and emission of one presenting frequency ω. The remaining term (B) are the delay-dependent term. The individual contributions are given by:

$$I_{l,r}(p) = \mathrm{e}^{\mathrm{i}(\Phi_l - \Phi_r)} P_{l,r} N(\epsilon_p) M_{l,r}(p), \tag{3}$$

$$P_{l,r} = \frac{\pi}{8} \int d\tau f_l(\tau) f_r(\tau) f_{IR}^2(\tau), \tag{4}$$

where τ indicates the time elapsed between the two interactions with the combined XUV-IR field. Time integrals are over all times, and the linearly-polarized light field is determined by carrier frequencies, XUV phases $\Phi_{1,2}$, and the field envelopes $f_{1,2}(t)$:

$$F_{IR}(t) = f_{IR}(t)\cos(\omega t), \tag{5}$$

$$F_i(t) = f_i(t)\cos(\Omega_i t + \Phi_i). \tag{6}$$

If the overall shape of the vibronic wavepacket, apart from the central position, is unaffected by nuclear motion, the average of the electronic matrix elements over the initial wave function

(Zero-point Energy, Z.P.E.) is:

$$M_{l,r}(p) \overset{\text{Z.P.E.}}{=} \sum_c G_{c,l,r}(p), \tag{7}$$

$$G_{c,l,r}(p) = \langle \chi_0(q) | \hat{D}^{\dagger}_{cp,0l}(q) \hat{D}_{cp,0r}(q) | \chi_0(q) \rangle, \tag{8}$$

where q indicates the nuclear coordinates. $N(\epsilon_p)$ is the Fourier transform of the autocorrelation function A (ε_p is the vibrational energy):

$$N(\epsilon_p) = \frac{1}{2\pi} \int d\tau \mathrm{e}^{+\mathrm{i}\epsilon_p \tau} A(\tau), \tag{9}$$

$$A(\tau) = \langle \chi_0(q) | \hat{u}^0(\tau) | \chi_0(q) \rangle, \tag{10}$$

$$\hat{u}^0(\tau) = \langle \psi(r;q) | \hat{U}_0(\tau) | \psi(r;q) \rangle, \tag{11}$$

$$\hat{U}_0(\tau) = \mathrm{e}^{-\mathrm{i}\int_0^\tau dt"\left(\hat{H}_0 - E_C\right)}, \tag{12}$$

where $\hat{H}_0$ is the field-free cationic Hamiltonian, and E_C is the energy origin, chosen as the vertical ionization energy. Finally, the two-photon ionization matrix elements $\hat{D}_{cp,a\pm}$ are given by the standard expressions:

$$\hat{D}_{cp,a\pm}(q) = \int dk \frac{\hat{\mu}_{p,ck}\hat{\mu}_{ck,a}}{(E_p - E_k \pm \omega) - \mathrm{i}0^+}, \tag{13}$$

$$\hat{\mu}_{p,ck}(q) = \langle \psi_p(r;q) | \hat{\mu}_{IR} | \psi_k(r;q) \rangle, \tag{14}$$

where $\hat{\mu}$ are the dipole-coupling operators.

For the most important special case, where $|\chi_0\rangle$ is the ground-state vibrational wavefunction of a multi-dimensional harmonic oscillator, the integral (8) can be readily evaluated. For a 1-dimensional harmonic oscillator of unit effective mass and force constant ω_i^2:

$$\left(-\frac{1}{2}\frac{\partial^2}{\partial q_i^2} + \frac{1}{2}\omega_i^2 q_i^2 - \frac{1}{2}\omega_i\right) \chi_{0,i}(q_i) = 0, \tag{15}$$

$$\chi_{0,i}(q_i) = \left(\frac{\omega_i}{\pi}\right)^{\frac{1}{4}} \mathrm{e}^{-\frac{\omega_i}{2} q_i^2}, \tag{16}$$

$$|\chi_0(q)\rangle = \prod_i \chi_{0,i}(q_i), \tag{17}$$

where q_i is the displacement from the equilibrium position $q_{0,i}$ and ω_i is the vibrational quantum. The classical turning points of the ground-state vibrational wavefunction of mode i are found at $q_i = \pm\omega_i^{-\frac{1}{2}}$. As long as the matrix elements $\hat{D}$ are sufficiently smooth and can be expanded in the Taylor series truncated at the second order, we obtain the final working expression:

$$G_{c,l,r}(p) \stackrel{Z.P.E.FD}{=} D_l^\dagger D_r + \sum_k D_l^\dagger W_{r,k} + \sum_k W_{l,k}^\dagger D_r + \sum_k V_{l,k}^\dagger V_{r,k} + 3\sum_k W_{l,k}^\dagger W_{r,k} + \sum_{k\neq m} W_{l,k}^\dagger W_{r,m}, \tag{18}$$

$$D_x = \hat{D}_{cp,0x}(q_c), \tag{19}$$

$$V_{x,k} = \frac{1}{\sqrt{8}}\left[\hat{D}_{cp,0x}^\dagger(q_0+\sqrt{\omega_k}) - \hat{D}_{cp,0x}^\dagger(q_0-\sqrt{\omega_k})\right], \tag{20}$$

$$W_{x,k} = \frac{1}{4}\left[\hat{D}_{cp,0x}(q_0+\sqrt{\omega_k}) + \hat{D}_{cp,0x}(q_0-\sqrt{\omega_k}) - 2\hat{D}_{cp,0x}(q_0)\right]. \quad , \tag{21}$$

where the $Z.P.E.FD$ indicate the average over the initial wave function using the standard finite-difference formulae. In Eqs. (18)–(21), q_c is the "characteristic" geometry (see below), q_0 is the equilibrium geometry, and the displacements $\pm\sqrt{\omega_k}$ correspond to the turning points of the zero-point wavefunction.

2.1 Technical details of the calculations

This section details more technical aspects of the calculations and spectral simulations, including construction of the vibronic Hamiltonian, evaluation of auto- and cross-correlation functions, treatment of photoionization matrix elements (1- and 2-photon), implementation of the zero-point energy correction, and spectral simulations.

2.2 Vibronic Hamiltonian

The quadratic vibronic Hamiltonian in the vicinity of the neutral equilibrium geometry is adopted from (16). Briefly, the T_d geometry and harmonic forcefield of neutral CH_4 were obtained at the MP2(fc)/aug-cc-pVTZ (52, 53) level. The quadratic vibronic Hamiltonian (54) for the $^2T_2$

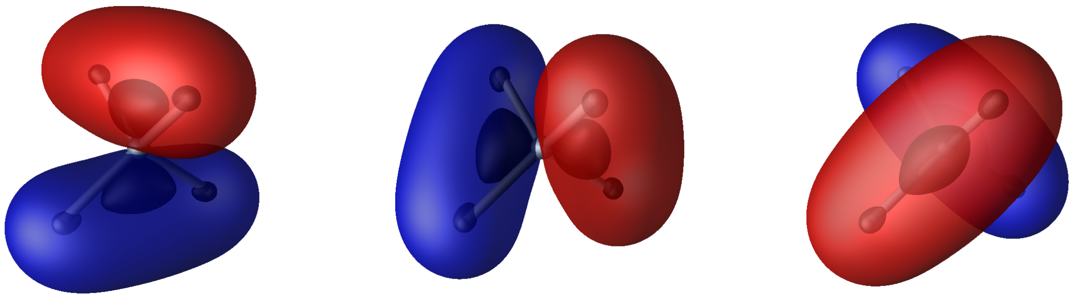

(a) (b) (c)

Figure S2: Dyson orbitals for the diabatic ionization channels in CH_4, T_d geometry, in the D_2 setting. Axes orientation is the same as in Fig. S3. The isosurface levels are ± 0.07 (outer) and ± 0.2 (inner) Bohr$^{-3/2}$. The positive and negative values are indicated by red and blue, respectively. a) B_1 ("Z") ionization channel; b) B_2 ("Y"); c) B_3 ("X").

cationic manifold was obtained by diabatization of MR-CIS energies in the vicinity of the neutral equilibrium, with CASSCF(7,4) reference averaged over the 3 lowest cation states. The resulting diabatic surfaces transform as the B_1 ("Z"), B_2 ("Y"), and B_3 ("Z") irreducible representations of the D_2 subgroup. The overall shapes of the Dyson orbitals corresponding to each of the diabatic ionization channels are illustrated in Fig. S2.

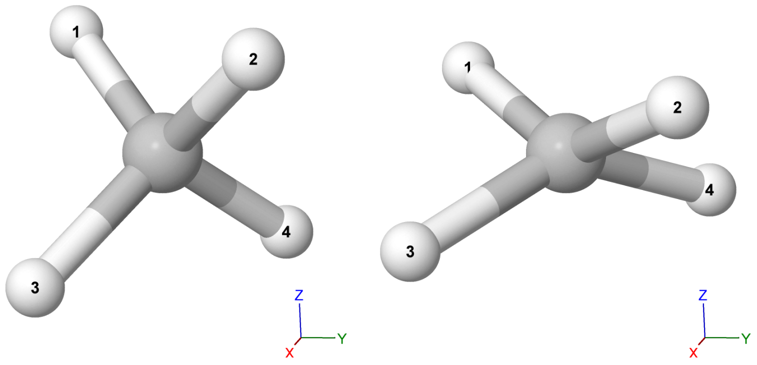

(a) (b)

Figure S3: Key structure for CH_4 and CH_4^+. (a) Equilibrium geometry of neutral CH_4 (T_d). (b) Stationary point on CH_4^+ B_1 ("Z") diabatic surface (D_2); full point group D_{2d}.

Table S1: Key structure parameters for CH_4 and CH_4^+ geometries; see Fig. S3.

	R_{CH} (Å)	$\alpha_{H_1CH_2}$ (deg)	$\alpha_{H_1CH_3}$ (deg)
CH_4 T_d	1.0862	109.47	
CH_4^+ D_{2d} min [1]	1.1190	139.16	96.98
CH_4^+ D_{2d} eff [2]	1.0954	111.15	108.64
CD_4^+ D_{2d} eff [3]	1.0918	110.47	108.97

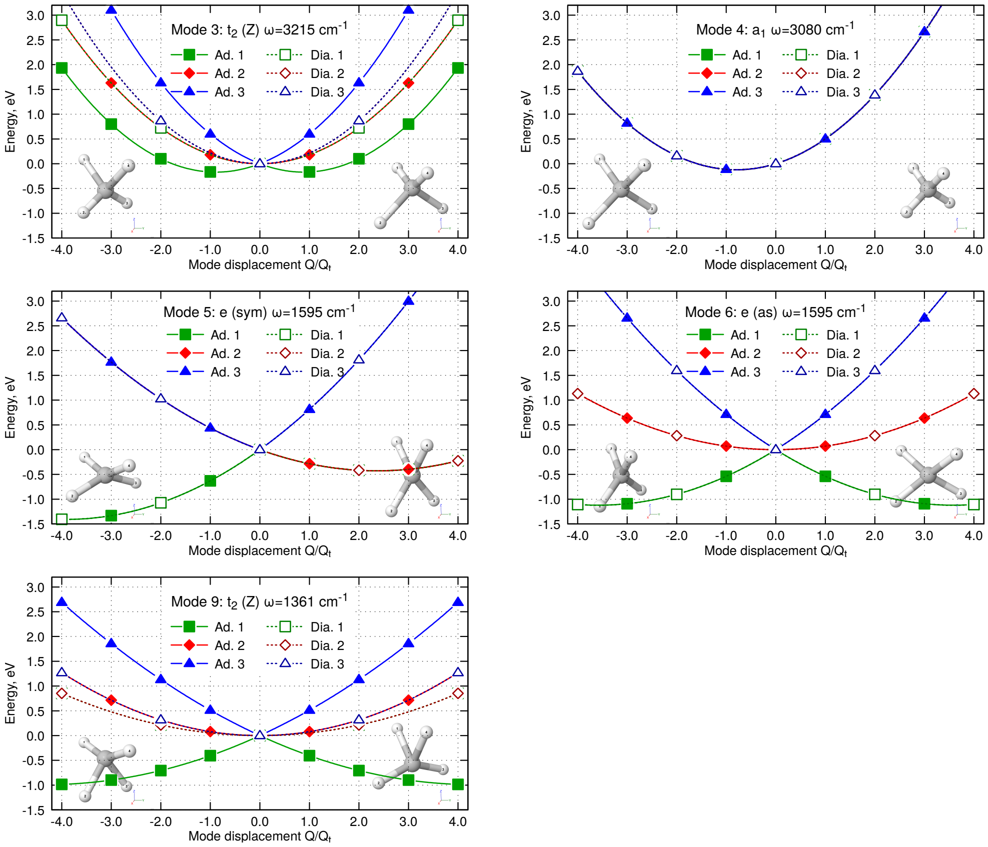

Figure S4: Cuts of the CH_4^+ T_2 potential energy surfaces along normal modes of neutral CH_4. Modes 1 and 2 are symmetry-equivalent to mode 3. Modes 7 and 8 are equivalent to mode 9. The displacements Q are shown as a fraction of the corresponding turning-point displacement for the zero-point mode of the neutral. The energies are relative to the full-symmetry position $Q = 0$, which possesses T_d symmetry. Adiabatic state energies are shown with solid lines and filled symbols. Diabatic states are indicated by dotted lines and open symbols.

Table S2: Autocorrelation simulation parameters.

$n_{\max}$[1]	N_{bas}[2]	$T_{\max}$[3], CH_4^+	$T_{\max}$[3], CD_4^+
2	2.2×10^3	60	75
4	1.8×10^5	110	125
8	1.2×10^9	210	235
12	7.6×10^{12}	300	320
16	5.0×10^{16}	> 300	> 320

The neutral T_d geometry and the stationary points on the D_2 diabatic surfaces, found about 1.41 eV below the vertical ionization energy, are illustrated in Fig. S3 and Table S1. We note that on the time scales relevant for the RABBIT experiment, only the immediate vicinity of the neutral equilibrium geometry is explored by the nuclear wavepacket (see below).

The shape of the cationic potential energy surface is illustrated by the cuts along the normal modes of the CH_4 neutral molecule, shown in Fig. S4. The full-symmetry point is unstable with respect to *all* normal-mode displacements. Distortion along the a_1 mode (#4) preserves the T_d symmetry. Distortions along the components of the e mode (#5, #6) lowers symmetry to D_2/D_{2d}, while the t_2 modes (#1–#3 and #7–#9) lower the symmetry to C_{2v}.

2.3 Evaluation of the autocorrelation function

Vibronic wave packets used to evaluate auto- and cross-correlation functions evolve on the vibronically-coupled diabatic electronic surfaces, comprising the $^2T_2$ cationic manifold. The simulation is full-dimensional, with the basis set spanning all three electronic surfaces and 9 vibrational modes of the reference harmonic surface. Excitations with up to 16 vibrational quanta in each normal mode are included in the simulation. The Hamiltonian H_0 includes all terms linear and quadratic in the normal-mode coordinates of the reference harmonic surface, between all pairs of diabatic electronic sufraces. The residual terms containing derivatives along the normal coordinates, remaining after the diabatization procedure, are neglected. The harmonic surface of the neutral species, at its equlibrium geometry, is used as the reference

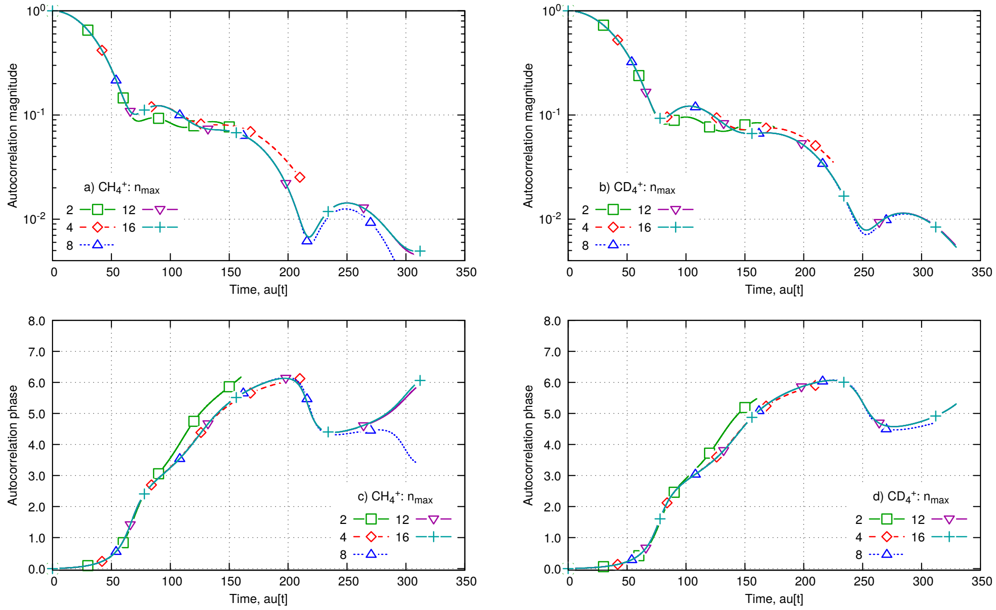

Figure S5: Convergence of autocorrelation function in Eq. (9) **with the basis set size.** Top row: Magnitude of autocorrelation function; bottom row: the phase of autocorrelation function, for CH_4^+ (left column) and CD_4^+ (right column). The individual curves are continued up to 100 au (≈ 2.4 fs) past the point where they diverge from the result of the more accurate simulation (See Table S2).

surface. The overall shape of the resulting PES is illustrated by the cuts along the normal-mode coordinates, given in Fig. S4. It should be emphasized that the initially-prepared vibronic wave packet in the cation evolves on the fully-coupled set of the diabatic surfaces, with all three surfaces becoming populated after a few femtoseconds (16,39,40). For symmetry reasons, only the vibrational component on the initially-populated diabatic surface contributes to the RABBIT signal in methane.

The numerical approach for evaluating the autocorrelation function $\langle \chi_0 | \hat{u}^0 (\tau; E_C) | \chi_0 \rangle$ in (9) follows Refs. (16, 55). Briefly, the cation's vibronic wavefunctions within the T_2 manifold are expanded in the product basis of the diabatic electronic functions and harmonic vibrational modes of the neutral species at the equilibrium geometry. Harmonic zero-point vibrational wavefunction of the neutral species is used as the initial wave packet at $t = 0$. It is propagated using 4th-order Runge-Kutta integrator, with the uniform time step of 0.02 atomic units of time (≈ 0.484 as). Amplitudes with magnitudes below 10^{-5} are forced to zero, to maintain sparsity of the wave packet representation. The parameters of the autocorrelation simulations are summarized in Table S2. The general treatment of the nuclear-motion effects (see Eq. (31) of ref. (35)) contains additional terms, beyond those given by the Eqs. (1),(2),(3),(4),(5),(6),(7),(8),(9) of the SM. These contributions contain time-dependent overlaps of a vibronic wave packet prepared on one diabatic surface at time t', and a wave packet placed on a different diabatic electronic surface at time t'' (the two-time cross-correlation function). Due to the high symmetry of the CH_4^+ cation, these contributions vanish for the vibronic wave packets derived from the vibrational ground state of the neutral species. The cross-correlation functions were evaluated in our vibronic-dynamics simulations, and were found to be negligibly small, as expected. Autocorrelation functions are compared in Fig. S5

We evaluate the Fourier transform in Eq. (9) using $n_{\max} = 16$ autocorrelation functions, truncated at $T_{\max} = 300$ au ($\approx$ 7.26 fs). The magnitude of the autocorrelation function is below

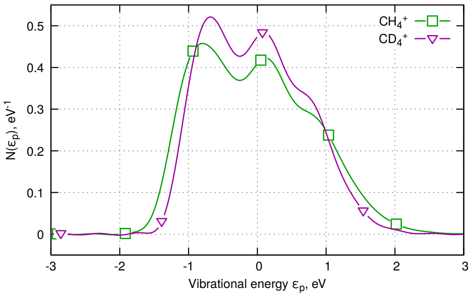

Figure S6: Fourier transform of the autocorrelation functions. Fourier transform of the autocorrelation functions for CH_4^+ (green line) and CD_4^+ (purple line) [Eq. (9)]. Vibrational structure below 0.3 eV is not resolved due to the truncation of the autocorrelation function in time, see text. Vibrational energy ϵ_p is with respect to the vertical transition from the CH_4/CD_4 neutral equilibrium geometry.

7×10^{-3} at this time, both for CH_4^+ and for CD_4^+. Substantial revivals do not occur until much longer times (40), and do not affect the overall shape of the $N(\epsilon_p)$ of Eq. (9). However, due to the truncation, we do not expect any vibrational structure below ≈ 0.3 eV (≈ 2300 cm^{-1}) to be resolved.

For evaluating the Fourier transform in Eq. (9), we extend the autocorrelation function to negative times using the relation $A(-\tau) = A(\tau)^\dagger$, and pad the result with zeros to $\approx \pm 414$ fs. The comparison of the resulting $N(\epsilon_p)$ functions is found in Fig. S6. Due to the time-reversal symmetry, the factor $N(\epsilon_p)$ is purely real. For an exact autocorrelation function, N is positive semidefinite. Time truncation introduces "ringing" in the outer parts of the numerical N (56), which could therefore become negative. The violation of the positivity does not exceed 0.5% of the total range of N, and is inconsequential for our purposes. The area under the curves in Fig. S6 is numerically found to be $1 \pm 2 \times 10^{-4}$, with 1 being expected analytically (56).

2.4 Evaluation of the characteristic geometries

We *estimate* the effects of the nuclear displacements on the electronic matrix elements in two steps: First we determine the characteristic evolution time t_{CH_4} (t_{CD_4}), defined as the ratio of the

first and zeroth momenta of the modulus of the nuclear autocorrelation function A (Eq. (10); Fig. S5):

$$t_c = \int_0^{t_{\max}} d\tau\tau \left|A\left(\tau\right)\right| / \int_0^{t_{\max}} d\tau \left|A\left(\tau\right)\right|, \tag{22}$$

where we use $t_{\max} = 300$ au (≈ 7.26 fs). The resulting characteristic times are 48.11 and 52.37 au (≈ 1.16 fs and ≈ 1.27 fs), respectively for CH_4 and CD_4.

We then estimate the expected geometry change at the characteristic time, by performing classical molecular dynamics on one of the three diabatic potential energy surfaces, up to the characteristic time t_c. The resulting structures, which have D_{2d} point-group symmetry, are summarized in Table S1. The distortions are qualitatively similar to those leading to the diabatic stationary points (see Fig. S3b): the C$-$H bonds are slightly elongated, accompanied by the opening of the H$-$C$-$H angles, pointing toward each of the lobes of the Dyson orbital for the diabatic ionization channel (see Figs. S2,S3).

We note that the D_{2d} diabatic structures are known to be unstable with respect to adiabatic symmetry-lowering along the q_7–q_9 t_2 modes (see Fig. S4), leading to C_{2v} global minima (39, 57). These dynamics, however, occur at time scales longer than those relevant to the present discussion. The short-time dynamics occurs primarily on the diabatic surfaces and preserves D_2 symmetry (39).

Due to their *ad hoc* definition, these "characteristic" geometries should be treated with extreme caution. In particular, the changes in the photoelectron spectra due to these distortions provide qualitative hints of the direction and possible magnitude of the effects, but are not to be treated quantitatively.

2.5 Photoionization matrix elements

The photoelectron matrix elements are computed as an expansion over tesseral harmonics ($L_{\max} = 6$), on a uniformly-spaced energy grid. Here, we only assess the quality of the matrix

elements by comparing them to the experimental 1-photon ionization cross-sections (58–60) and He(I) photoelectron spectra (26, 61) of CH_4. Unfortunately, no comparable reference compendium appears to be available for CD_4.

Through the comparison of the calculated and measured photoelectron spectra (below), we fix the vertical ionization potential of CH_4 to vIP $= 14.4$ eV. This value is somewhat higher than the 13.6 eV value, usually assumed in the literature, but leads to a better overall agreement with the experiment.

Neglecting the zero-point effects, the angle- and energy-resolved, laboratory-frame one-photon ionization cross-sections in a_0^2 per Hartree and steradian are calculated as:

$$\sigma\left(E_p, \vec{n}_{E_p}; \Omega, \vec{n}_\Omega\right) = \frac{4\pi^2}{c}\Omega \sum_a N_a\left(\epsilon_p\right) \iiint d\alpha d\beta d\gamma \left| d_a\left(E_p, \vec{n}_{E_p}, \vec{n}_\Omega; \alpha, \beta, \gamma\right)\right|^2, \qquad (23)$$

where c is the speed of light, Ω is the photon energy, E_p is the photoelectron energy at the detector, $\vec{n_{E_p}}$ and $\vec{n_\Omega}$ are molecular-frame direction to the detector and molecular-frame XUV polarization direction. Finally, $\epsilon_p = \Omega - (E_p + \text{vIP})$ is the vibrational energy (we note that ϵ_p can become negative). The sum over a runs over all three diabatic cation states. The integration is over the full domain of the Euler angles describing the molecular orientation in the laboratory frame.

The energy-resolved, angle-integrated cross-section [the photoelectron spectrum (PES)] and the angle-resolved photoionization cross-section are obtained by additionally integrating $\sigma\left(E_p, \vec{n}_{E_p}; \Omega, \vec{n}_\Omega\right)$:

$$\sigma\left(E_p; \Omega\right) = \iint d\vec{n}_{E_p} \sigma\left(E_p, \vec{n}_{E_p}; \Omega, \vec{n}_\Omega\right), \qquad (24)$$

$$\sigma\left(\Omega, \theta\right) = \int dE_p \sigma\left(E_p, \vec{n}_{E_p}; \Omega, \vec{n}_\Omega\right)\Big|_{\vec{n}_{E_p}\cdot\vec{n}_\Omega = \cos\theta}, \qquad (25)$$

where in Eq. (24), dependence on $\vec{n}_\Omega$ drops out due to the rotational isotropy of the free space. In Eq. (25), electrons are observed at angle θ from laser polarization direction $\vec{n}_\Omega$. The energy-

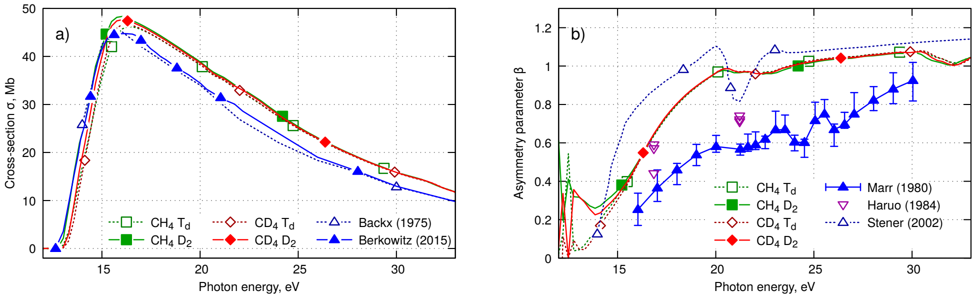

Figure S7: One-photon ionization cross-sections σ and asymmetry parameter β. Measured vs. calculated one-photon ionization cross-sections σ for CH_4(a). Measured vs. calculated asymmetry parameters β for CH_4 (b). Experimental data are from Backx *et al* (58), Marr *et al* (59), Shiromaru *et al* (62), and Berkowitz (60). Calculated values (TD-DFT) are from Stener *et al* (63). Calculated values from Eq. 26, for the D_2 characteristic (solid green lines) and T_d neutral equilibrium (dotted lines) geometries.

resolved cross-section $\sigma\left(\Omega\right)$ and photoelectron asymmetry parameter $\beta\left(\Omega\right)$ are then given by:

$$\sigma\left(\Omega\right) = \sigma\left(\Omega, \theta_M\right), \tag{26}$$

$$\beta\left(\Omega\right) = \frac{\sigma\left(\Omega, 0\right)}{\sigma\left(\Omega, \theta_M\right)} - 1, \tag{27}$$

where $\theta_M = \arccos \frac{1}{\sqrt{3}}$ is the magic angle.

Calculated one-photon ionization cross-sections are shown in Fig. S7a, in comparison with the experimental cross-sections for CH_4. (Unfortunately, no experimental CD_4 cross-sections appear to be available in the literature.) The calculated cross-sections are systematically overestimated by $\approx 10\%$ compared to the experimental results (60), with somewhat larger deviations from the older results of Ref. (58). The agreement is otherwise remarkably good, considering that no empirical adjustments were applied to the numerical results. The difference between calculated CH_4 and CD_4 cross-sections are very small, with CD_4 cross-section being $\approx 1.5\%$ lower. Cross-sections calculated at the "characteristic" (D_2), rather than the equilibrium (T_d) geometry appear to be in a better agreement with experiment, with the position of the cross-section maximum moving to lower photoelectron energies.

The comparison of calculated and measured asymmetry parameters β is given in Fig. S7b.

The agreement with the measured β parameter (59) is at best qualitative: the overall trend of decreasing β for lower photon energies is reproduced, but the calculated numerical values are well outside the errorbars. It should be noted that the β values of Ref. (59) may be less accurate than claimed: The experimental values of Ref. (62), measured at the He(I) and Ne(I) wavelengths, are also outside of the claimed errorbars, and closer to the calculated values. On the other hand, apart from the missing feature at $\approx$ 22 eV, which may be associated with ionization from the $2a_1$ molecular orbital of CH_4 (not included in the present simulations), our calculated β values are in a pleasing agreement with the calculated TD-DFT asymmetry parameters from Ref. (63). Overall, we conclude that our theoretical approach is adequate for describing the gross features of one-photon ionization of CH_4.

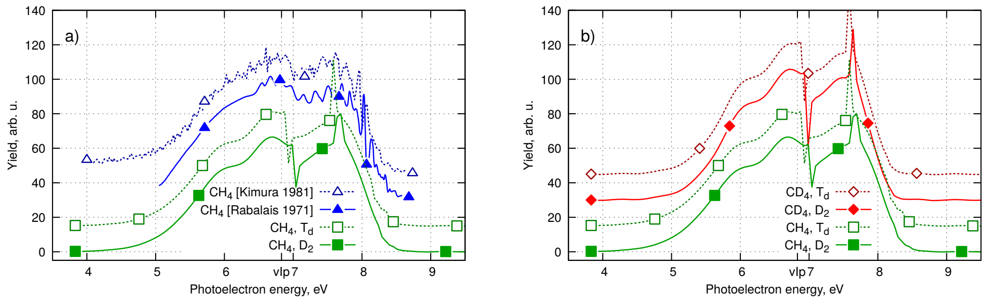

Figure S8: Measured and calculated photoelectron spectra for CH_4 and CD_4. a) Measured and calculated He(I) photoelectron spectra for CH_4. Experimental data from Kimura *et al* (61) and Rabalais *et al* (26). The spectra are shifted vertically to improve readability. Please note that vibrational structure below 0.3 eV is not resolved on the calculated spectra, see text. Sharp features in the calculated spectra are due to electronic resonances. b) Calculated CH_4 vs CD_4 He(I) spectra.

We further compare simulated and experimental results for the He(I) photoelectron spectra (21.23 eV light) in Fig. S8a. Due to the truncation of the autocorrelation function at $\approx$ 7.26 fs, vibrational structure below 0.3 eV is not resolved in the simulated spectra. The overall shape of the PES is however very well reproduced, with two, broad main peaks at $\approx$ 7.6 eV and 6.7 eV, and a broad shoulder at $\approx$ 6.1 eV (expt: 7.6, 6.8, and 6.2 eV (61)). Two electronic

resonances appear in the simulated spectra, so that the fine structure in experiment is likely due to the interplay of vibrational and electronic dynamics. The simulated CD_4 He(I) PES is very slightly narrower (Fig. S8b), with the three peaks at ≈ 7.5 eV, 6.7 eV, and 6.2 eV, and noticeably compressed shoulders on both sides. Unlike for the cross-section σ, accounting for structure relaxation does not noticeably improve agreement with the experiment (D_2 vs. T_d).

2.6 Finite-difference realization of the zero-point contributions

The photoelectron matrix elements, both one- and two-photon, are presently only available at fixed geometries. In order to evaluate the Z.P.E. corrections to the 2-photon RABBIT matrix elements using the lowest-order finite-difference scheme [Eq. (18)], we require two displacements per normal mode, in addition to the central, equilibrium geometry. For CH_4 and CD_4, which possess 9 normal modes, we would therefore require 18 distorted geometries per isotopomer. The resulting calculation would be prohibitively expensive, both due to the number of the structures and their reduced point-group symmetry. Using molecular symmetry, we can reduce this number to just 7 unique distorted geometries, with the remaining 11 structures, and the corresponding matrix elements, obtained by symmetry-transforming the results.

For the t_2 vibrational modes, we require the distorted geometries to be symmetric with respect to the C_{2x}, C_{2y}, or C_{2z} symmetry element in the standard setting of the T_d point group (64). A single, unique geometry per degenerate set is then sufficient. For the e modes, we choose distortions to be either symmetric or anti-symmetric with respect to the σ_{d2} plane (64) [symmetry plane normal to the $(1, -1, 0)$ Cartesian direction]. Three unique geometries are needed per e degenerate set. Finally, the fully-symmetric, non-degenerate a_1 modes require two displacements per mode. The distorted structures are illustrated in Figs. S9 and S10. The overall effect on the nuclear probability distribution is also illustrated in Fig. S11.

An additional slight complication arises during evaluation of the Z.P.E. corrections to the

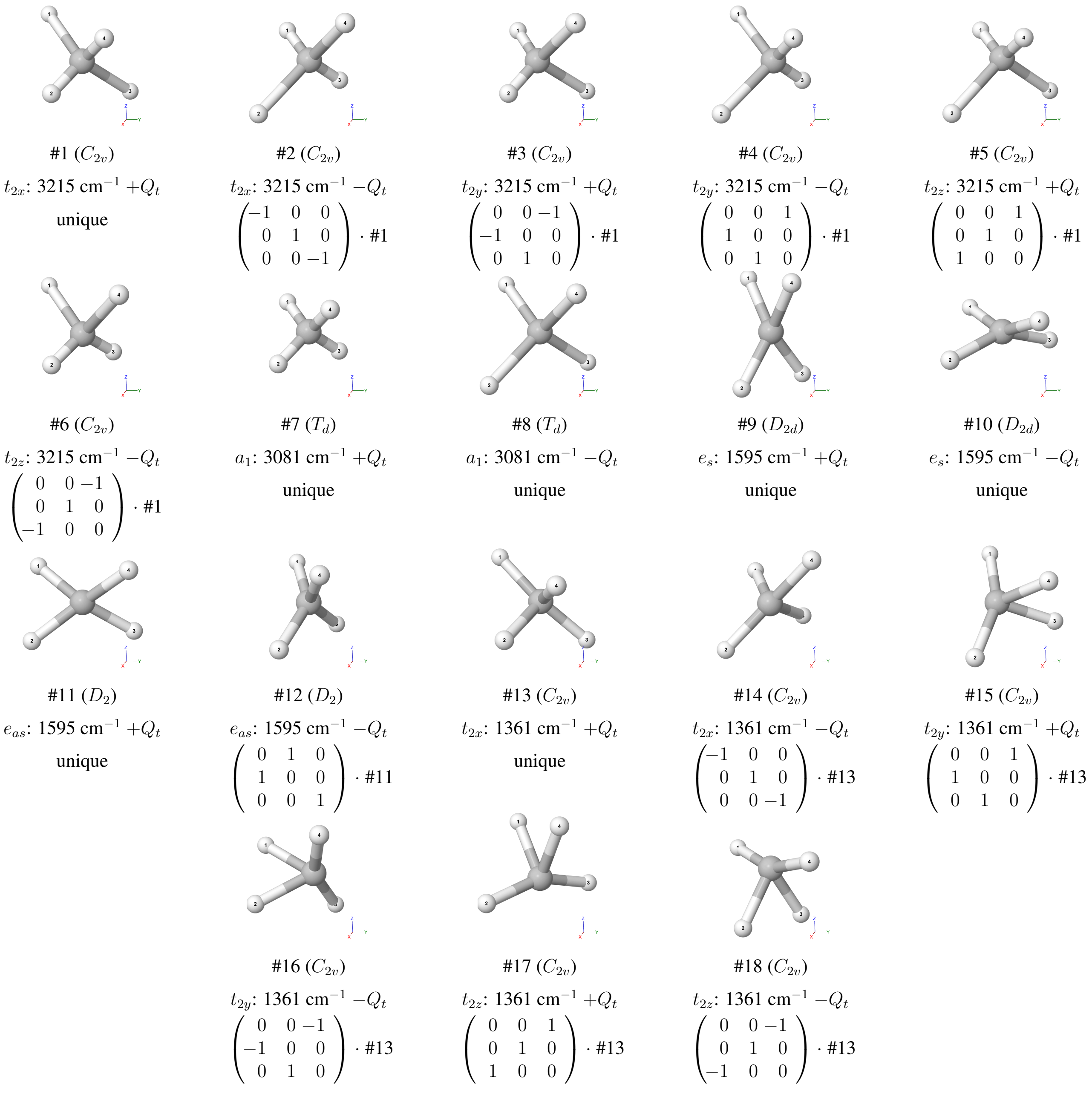

Figure S9: Distorted geometries used for evaluating Z.P.E. correction to 2-electron integrals in CH_4. Two distorted structure per normal mode is shown, one each for the outer ($+Q_t$) and inner ($-Q_t$) turning points of the vibrational ground state. For visibility, magnitude of the distortion are exaggerated by a factor of $5\times$. Some of the structures are symmetry-equivalent; the corresponding coordinate transformation matrix and the reference structure are listed below the structure.

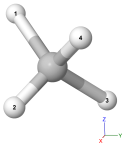

#1 (C_{2v})

t_{2x}: 2379 cm^{-1} $+Q_t$

unique

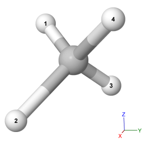

#2 (C_{2v})

t_{2x}: 2379 cm^{-1} $-Q_t$

$\begin{pmatrix} -1 & 0 & 0 \\ 0 & 1 & 0 \\ 0 & 0 & -1 \end{pmatrix} \cdot$ #1

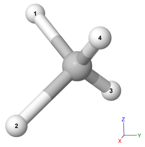

#3 (C_{2v})

t_{2y}: 2379 cm^{-1} $+Q_t$

$\begin{pmatrix} 0 & 0 & 1 \\ 1 & 0 & 0 \\ 0 & 1 & 0 \end{pmatrix} \cdot$ #1

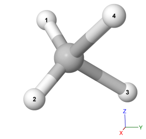

#4 (C_{2v})

t_{2y}: 2379 cm^{-1} $-Q_t$

$\begin{pmatrix} 0 & 0 & -1 \\ -1 & 0 & 0 \\ 0 & 1 & 0 \end{pmatrix} \cdot$ #1

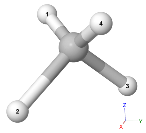

#5 (C_{2v})

t_{2z}: 2379 cm^{-1} $+Q_t$

$\begin{pmatrix} 0 & 0 & 1 \\ 0 & 1 & 0 \\ 1 & 0 & 0 \end{pmatrix} \cdot$ #1

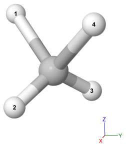

#6 (C_{2v})

t_{2z}: 2379 cm^{-1} $-Q_t$

$\begin{pmatrix} 0 & 0 & -1 \\ 0 & 1 & 0 \\ -1 & 0 & 0 \end{pmatrix} \cdot$ #1

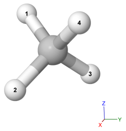

#7 (T_d)

a_1: 2178 cm^{-1} $+Q_t$

unique

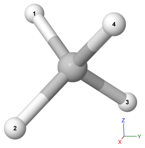

#8 (T_d)

a_1: 2178 cm^{-1} $-Q_t$

unique

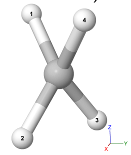

#9 (D_{2d})

e_s: 1128 cm^{-1} $+Q_t$

unique

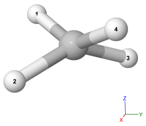

#10 (D_{2d})

e_s: 1128 cm^{-1} $-Q_t$

unique

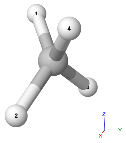

#11 (D_2)

e_{as}: 1128 cm^{-1} $+Q_t$

unique

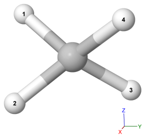

#12 (D_2)

e_{as}: 1128 cm^{-1} $-Q_t$

$\begin{pmatrix} 0 & 1 & 0 \\ 1 & 0 & 0 \\ 0 & 0 & 1 \end{pmatrix} \cdot$ #11

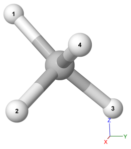

#13 (C_{2v})

t_{2x}: 1028 cm^{-1} $+Q_t$

unique

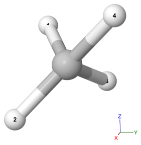

#14 (C_{2v})

t_{2x}: 1028 cm^{-1} $-Q_t$

$\begin{pmatrix} -1 & 0 & 0 \\ 0 & 1 & 0 \\ 0 & 0 & -1 \end{pmatrix} \cdot$ #13

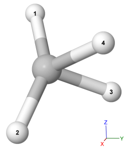

#15 (C_{2v})

t_{2y}: 1028 cm^{-1} $+Q_t$

$\begin{pmatrix} 0 & 0 & 1 \\ 1 & 0 & 0 \\ 0 & 1 & 0 \end{pmatrix} \cdot$ #13

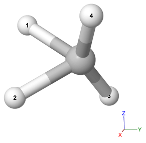

#16 (C_{2v})

t_{2y}: 1028 cm^{-1} $-Q_t$

$\begin{pmatrix} 0 & 0 & -1 \\ -1 & 0 & 0 \\ 0 & 1 & 0 \end{pmatrix} \cdot$ #13

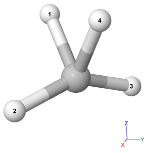

#17 (C_{2v})

t_{2z}: 1028 cm^{-1} $+Q_t$

$\begin{pmatrix} 0 & 0 & 1 \\ 0 & 1 & 0 \\ 1 & 0 & 0 \end{pmatrix} \cdot$ #13

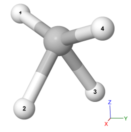

#18 (C_{2v})

t_{2z}: 1028 cm^{-1} $-Q_t$

$\begin{pmatrix} 0 & 0 & -1 \\ 0 & 1 & 0 \\ -1 & 0 & 0 \end{pmatrix} \cdot$ #13

Figure S10: Distorted Z.P.E. geometries in CD_4. See Fig. S9 caption.

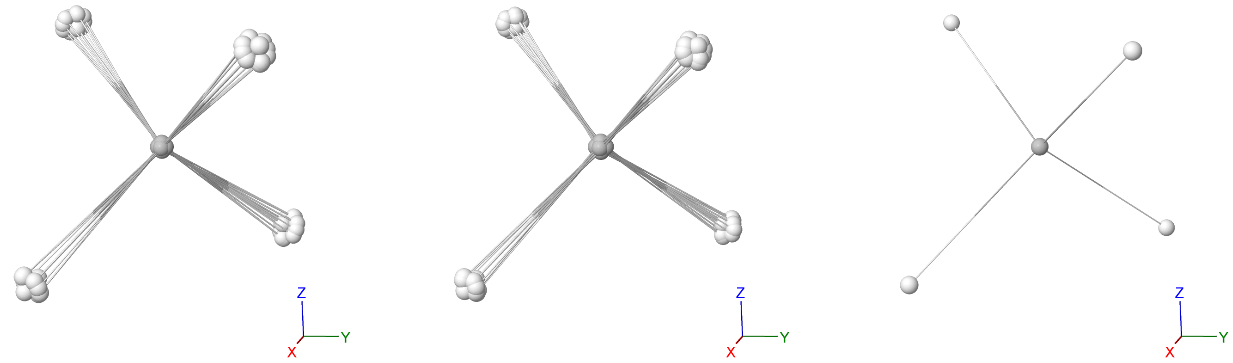

(a) CH_4 (b) CD_4 (c) frozen

Figure S11: Zero-point energy wavefunctions in CH_4 and CD_4. Nuclear configuration space accessed by the zero-point wavefunctions in CH_4 (a) and CD_4 (b). Sphere of a radius 0.05 Å is placed at the coordinates of each atom, for each turning point in Figs. S9, S10. Neutral equilibrium geometry is rendered in the same way for comparison (c).

matrix elements. The 2-photon matrix elements are evaluated for transition between *adiabatic* electronic states, while our theory is formulated in terms of the *diabatic* states. Furthermore, the phases of the initial and final states at different geometries are not guaranteed to be consistent. For our choice of the molecular geometry (the default setting of the T_d point group) and computational point group (D_2, both at the high-symmetry point and at the "characteristic" geometries), diabatic and adiabatic states coincide. It is therefore only necessary to diabatize the matrix elements at normal-mode turning points, before using Eq. (18).

We diabatize the matrix elements following the maximum-overlap approach (65–67). Briefly:

$$\hat{D}^{\mathrm{dia}}_{cp,0r}(q) = \sum_{d} \hat{D}^{\mathrm{ad}}_{dp,0r}(q)\, U_{dc}(q)\,, \tag{28}$$

$$\mathbf{U} = \left(\mathbf{S}\mathbf{S}^{\dagger}\right)^{-1/2} \mathbf{S}, \tag{29}$$

where $\hat{D}^{\mathrm{dia}}_{cp,0r}$ and $\hat{D}^{\mathrm{ad}}_{dp,0r}$ are the diabatic and adiabatic matrix elements, respectively. The matrix $\mathbf{S}$ is an overlap matrix between the Dyson orbitals of adiabatic states at the displaced geometry

(rows) and diabatic states at the reference geometry:

$$S_{ad}(q) = \langle \phi_a^{\text{Dyson,ad}}(q) | \phi_d^{\text{Dyson,dia}}(q_{\text{ref}}) \rangle , \tag{30}$$

$$|\phi_a^{\text{Dyson,ad}}(q)\rangle = \sqrt{N_e} \langle \psi_a^{\text{ad}}(q) | \psi_0(q) \rangle , \tag{31}$$

$$|\phi_d^{\text{Dyson,dia}}(q_{\text{ref}})\rangle = \sqrt{N_e} \langle \psi_d^{\text{dia}}(q_{\text{ref}}) | \psi_0(q_{\text{ref}}) \rangle , \tag{32}$$

where N_e is the number of electrons in the neutral species, and we assumed that adiabatic and diabatic wavefunctions of the neutral species coincide.

Additionally, symmetry transformation of the molecular geometry applies a unitary transform to the diabatic states within the degenerate manifold. The necessary transformation matrices are readily available (64). In the specific case of CH_4, we are interested in transforming the B_3, B_2, and B_1 irreducible representations of the D_2 subgroup of the the full symmetry group (T_d). These transformations are isomorphic with those of the X, Y, and Z Cartesian vectors.

We evaluate the Z.P.E. corrections to two-electron matrix elements from Eq. (18). Because the matrix elements D_x [Eq. (19)] depend on the choice of the central geometry (the full-symmetry T_d neutral equilibrium or "characteristic"), we need to precompute and store the $V_{x,k}$ and $W_{x,k}$ matrix elements [Eqs. (20) and (21)]. We use the same tesseral-harmonic expansion employed by the underlying photoelectron matrix elements. We, however, increase the $L_{\max}$ to 8, to account for the possible increase in angular-momentum content due to numerical differentiation.

2.7 Spectral simulations

For simulating the two-photon RABBIT spectra, we employ Eqs. (1) and (3). The nuclear part of the matrix elements is evaluated from Eq. (9). The electronic part is evaluated from (18) (including the Z.P.E. correction). The Fourier transforms of the autocorrelation function [Eq. (9)] are represented on a uniformly-spaced grid of ϵ_p (from -3 eV to $+3$ eV with 0.005 eV spac-

ing), interpolated with "natural" cubic splines (68). Electronic matrix elements are represented by tesseral-harmonic expansions ($L_{\rm max} = 6$ for the matrix elements at the equilibrium neutral and "characteristic" geometries; $L_{\rm max} = 8$ for the Z.P.E. corrections). The radial parts of the two-photon matrix elements is evaluated within ± 3 eV (0.1 eV increments) around the vertical sideband centre, found at $1.225n - 14.4$ eV. They are likewise interpolated by "natural" cubic splines. Finally, the Z.P.E. correction integrals are evaluated on a uniform radial grid between 0 eV and 20 eV, with 0.1 eV spacing, and cubic-spline interpolated. Only the values at the band centres are reliable for the Z.P.E. terms. These are applied to the entire band.

Some of the observables require orientational averaging of the molecular-frame signal, given by Eq. (1). Averaging over field-polarization directions is performed on the Lebedev grid (69) of the order 17, sufficient to integrate spherical harmonics with $L \leq 17$. The same grid is used for integrating over the first two Euler angles describing molecular orientation (α, β). The final Euler angle (γ) is integrated using 35-point uniform grid, which is of the same order. Integration over the photoelectron energy [in eq. (25)] is performed using an order-1000 Gauss-Legendre grid (70).

2.8 Model for constructing the two-color photoelectron spectra

The simulated XUV-only spectra were compared with the photoelectron spectra measured using monochromatic synchrotron radiation.

The simulated XUV-only spectra and the two-color photoelectron sidebands were spectrally split into the three contributions corresponding to photoelectrons associated to the CH_2^+-CD_2^+, CH_3^+-CD_3^+ and CH_4^+-CD_4^+ ionic channels. The spectral filter functions were adapted to the

experimental branching ratios extracted from ref. (33) and are given by:

$$\begin{aligned} F_{\mathrm{CH}_4^+} &= \exp[-(E-E_{N_1})^{10}/(2*1.63^{10})] \\ F_{\mathrm{CH}_3^+} &= 1-F_{\mathrm{CH}_4^+}-F_{\mathrm{CH}_2^+} \\ F_{\mathrm{CH}_2^+} &= 1-\exp[-(E-E_{N_2})^{10}/(2*2.72^{10})]\times 0.18. \end{aligned} \quad (33)$$

The centers of the filters E_{N_1} and E_{N_2} were adapted to the central energy positions of each harmonic and each sideband. The comparison between the experimental branching ratios and those described by Eq. 33 is presented in Fig. S1. The obtained photoelectron peaks were then convoluted with a Gaussian with 1000 meV FWHM that mimics the spectrometer resolution. This procedure is repeated for every harmonic and sideband peak.

A synthetic RABBIT scan was obtained by combining the theory data from the harmonics and sidebands. The oscillations of sideband n are calculated according to the expression:

$$I_n^{SB}(\Delta t) = GN\left(|A_{n-1}|^2 S_n^{+,+} + |A_{n+1}|^2 S_n^{-,-} + 2\mathrm{Re}\left[\exp(i2\omega\Delta t)|A_{n-1}||A_{n+1}|S_n^{+,-}\right]\right) \quad (34)$$

where N is the Fourier transform of the autocorrelation function, $|A_{n\pm1}|$ is the amplitude of the harmonics, $S_n^{\pm,\pm}$ are the two-photon matrix elements, and G indicates the filter(s) used to separate the dissociative (CH_3^+ and CH_2^+) and non-dissociative (CH_4^+) channels. The theory data for the harmonics are calculated in the absence of an IR field and is hence independent of delay. We keep the harmonic intensity constant as a function of delay and only modulate the sideband intensity with delay. The relative intensity of the different harmonics was adjusted by comparison using the experimental CH_4^+ photoelectron spectrum. The relative intensity between harmonics and sidebands was scaled (the scaling factor SF is the same for all harmonics: $\mathrm{SF} = 650000$) in order to reproduce the experimental data. We have verified that other scaling factors corresponding to a reasonable agreement between simulated and experimental two-color photoelectron spectra ($450000 < \mathrm{SF} < 850000$), determine a similar evolution of the parame-

ters $A_{0\omega}$, $A_{2\omega}$, C and $\Delta\varphi$ as a function of the sideband order, indicating the robustness of our approach and of the numerical analysis.

Figure S12 presents the comparison between the channel-resolved experimental and simulated delay-integrated RABBIT spectrogram in methane and deuteromethane. The agreement between the curves is good, supporting the application of our approach for the simulations of the two-color photoionization experiment. The delay-resolved comparison of the RABBIT traces is presented in Fig. S13.

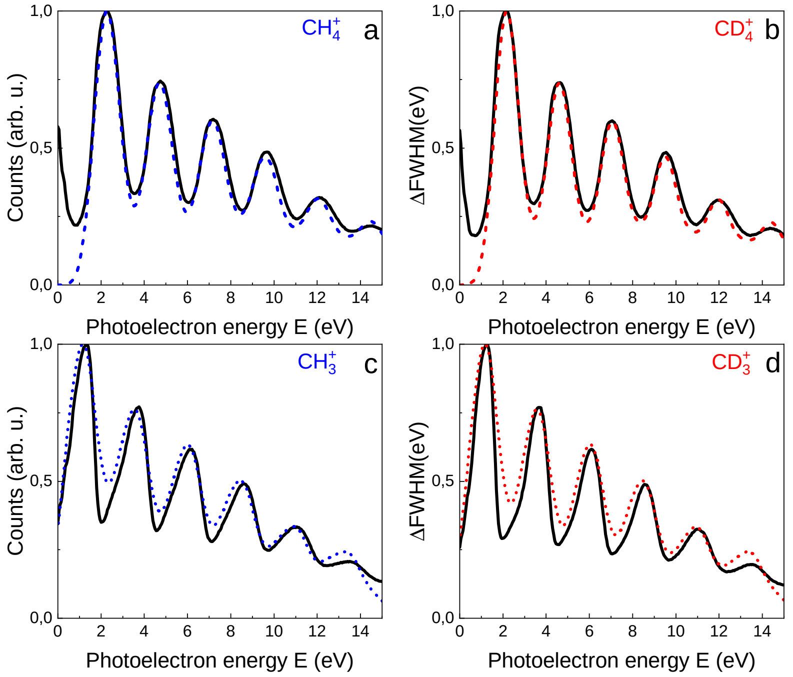

Figure S12: Simulated channel-resolved and delay-integrated RABBIT spectrograms in methane and deuteromethane. Comparison between experimental (black solid lines) and simulated spectra (blue and red lines) measured in coincidence with the ionic fragments CH_4^+ (a) (blue dashed line), CH_3^+ (b) (blue dotted line), CD_4^+ (c) (red dashed line), and CD_3^+ (d) (red dotted line). The experimental and simulated photoelectron spectra were integrated over the delay Δt between the attosecond pulse train and the IR pulse.

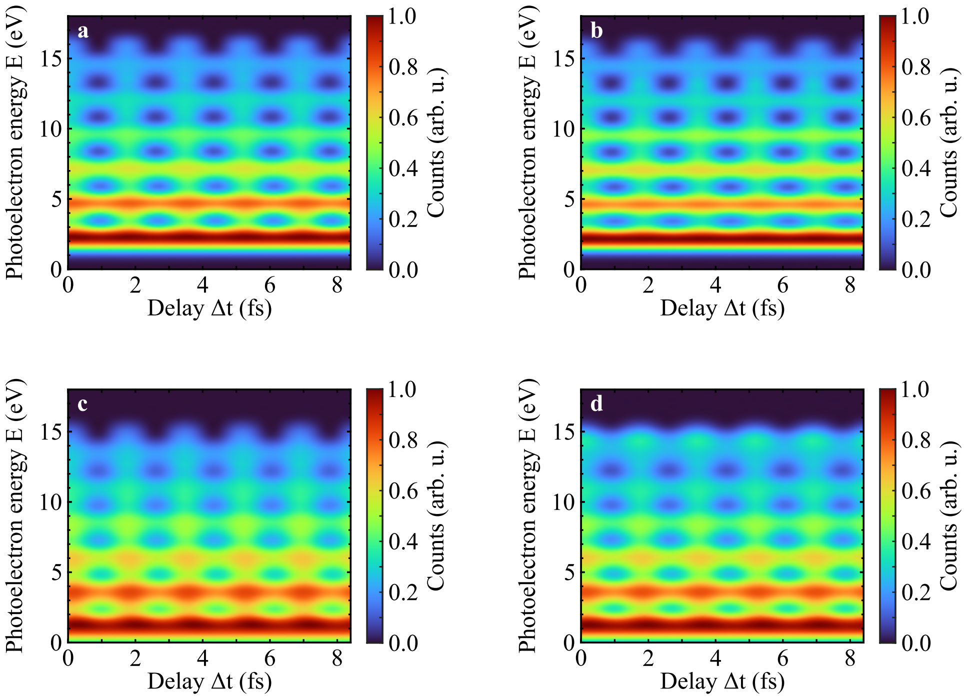

Figure S13: Simulated channel-resolved RABBIT spectrograms in methane and deuteromethane. Simulated RABBIT traces obtained considering only the photoelectrons associated with the ion CH_4^+ (a), CD_4^+ (b), CH_3^+ (c), and CD_3^+ (d). In the simulation, the effect of the attosecond chirp was not included. Adapted with permission from ref. (48).

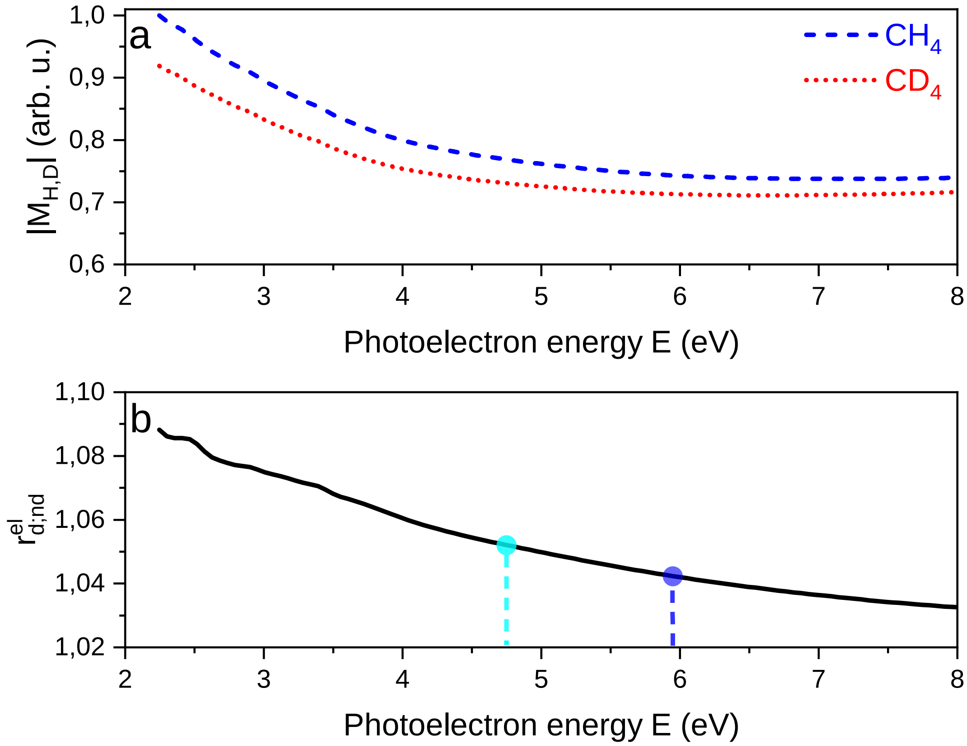

Figure S14: Electronic part of the matrix dipole moment. (a) Matrix dipole element for the electronic part for CH_4 (blue line) and CD_4 (red line) for the energies corresponding to the SB16. (b) Ratio $r^{el}_{d,nd}$ of the electronic matrix dipole moment for CH_4 and CD_4 as a function of the photoelectron energy. The light-blue and blue circles indicate the centers of the SB16 associated to the dissociating (CH_3^+-CD_3^+) and non dissociating channels (CH_4^+-CD_4^+), respectively.

Table S3: Electronic and nuclear contributions to the ratios of the sideband oscillations for dissociating and non-dissociating channels. Comparison of the ratios $\mathrm{r_{d,nd}}$ obtained from the experimental data (first line) and from the numerical theoretical model (second line). The third and fourth lines present the contribution of the electronic ($\mathrm{r^{el}_{d,nd}}$) and nuclear ($\mathrm{r^{nucl}_{d,nd}}$) ratios to the final value $\mathrm{r_{d,nd}}$=$\mathrm{r^{el}_{d,nd}} \cdot \mathrm{r^{nucl}_{d,nd}}$ (see Eq. 3 of main manuscript).

	Ratio $\mathrm{r_{nd}}$ $(\mathbf{CH_4^+} - \mathbf{CD_4^+})$			
SB order	14	16	18	20
Exp.	0.917 ± 0.087	0.945 ± 0.079	0.911 ± 0.069	0.909 ± 0.14
Theory	1.017 ± 0.046	1.009 ± 0.055	1.000 ± 0.052	1.004 ± 0.071
$\mathrm{r^{el}_{nd}}$	1.067	1.042	1.027	1.018
$\mathrm{r^{nucl}_{nd}}$	1.017	1.017	1.017	1.017
$\mathrm{r_{nd}}$	1.085	1.060	1.044	1.036

	Ratio $\mathrm{r_{d}}$ $(\mathbf{CH_3^+} - \mathbf{CD_3^+})$			
SB order	14	16	18	20
Exp.	0.831 ± 0.109	0.784 ± 0.086	0.800 ± 0.094	0.802 ± 0.089
Theory	1.023 ± 0.033	0.948 ± 0.028	0.924 ± 0.033	0.923 ± 0.029
$\mathrm{r^{el}_{d}}$	1.128	1.052	1.030	1.020
$\mathrm{r^{nucl}_{d}}$	0.894	0.894	0.894	0.894
$\mathrm{r_{d}}$	1.008	0.940	0.921	0.912

REFERENCES AND NOTES

1. G. Sansone, T. Pfeifer, K. Simeonidis, A. I. Kuleff, Electron correlation in real time. *ChemPhysChem* **13**, 661–680 (2012).

2. M. Nisoli, P. Decleva, F. Calegari, A. Palacios, F. Martín, Attosecond electron dynamics in molecules. *Chem. Rev.* **117**, 10760–10825 (2017).

3. E. Goulielmakis, Z.-H. Loh, A. Wirth, R. Santra, N. Rohringer, V. S. Yakovlev, S. Zherebtsov, T. Pfeifer, A. M. Azzeer, M. F. Kling, S. R. Leone, F. Krausz, Real-time observation of valence electron motion. *Nature* **466**, 739–743 (2010).

4. H. Niikura, F. Légaré, R. Hasbani, M. Y. Ivanov, D. M. Villeneuve, P. B. Corkum, Probing molecular dynamics with attosecond resolution using correlated wave packet pairs. *Nature* **421**, 826–829 (2003).

5. M. Meckel, D. Comtois, D. Zeidler, A. Staudte, D. Pavičić, H. C. Bandulet, H. Pépin, J. C. Kieffer, R. Dörner, D. M. Villeneuve, P. B. Corkum, Laser-induced electron tunneling and diffraction. *Science* **320**, 1478–1482 (2008).

6. B. Wolter, M. G. Pullen, A.-T. Le, M. Baudisch, K. Doblhoff-Dier, A. Senftleben, M. Hemmer, C. D. Schröter, J. Ullrich, T. Pfeifer, R. Moshammer, S. Gräfe, O. Vendrell, C. D. Lin, J. Biegert, Ultrafast electron diffraction imaging of bond breaking in di-ionized acetylene. *Science* **354**, 308–312 (2016).

7. M. Lara-Astiaso, A. Palacios, P. Decleva, I. Tavernelli, F. Martín, Role of electron-nuclear coupled dynamics on charge migration induced by attosecond pulses in glycine. *Chem. Phys. Lett.* **683**, 357–364 (2017).

8. M. Vacher, M. J. Bearpark, M. A. Robb, J. A. P. Malhado, Electron dynamics upon ionization of polyatomic molecules: Coupling to quantum nuclear motion and decoherence. *Phys. Rev. Lett.* **118**, 083001 (2017).

9. M. Lein, Attosecond probing of vibrational dynamics with high-harmonic generation. *Phys. Rev. Lett.* **94**, 053004 (2005).

10. S. Baker, J. S. Robinson, C. A. Haworth, H. Teng, R. A. Smith, C. C. Chirilă, M. Lein, J. W. G. Tisch, J. P. Marangos, Probing proton dynamics in molecules on an attosecond time scale. *Science* **312**, 424–427 (2006).

11. S. Haessler, W. Boutu, M. Stankiewicz, L. J. Frasinski, S. Weber, J. Caillat, R. Taïeb, A. Maquet, P. Breger, P. Monchicourt, B. Carré, P. Salières, Attosecond chirp-encoded dynamics of light nuclei. *J. Phys. B At. Mol. Opt. Phys.* **42**, 134002 (2009).

12. S. Patchkovskii, Nuclear dynamics in polyatomic molecules and high-order harmonic generation. *Phys. Rev. Lett.* **102**, 253602 (2009).

13. D. R. Austin, A. S. Johnson, F. McGrath, D. Wood, L. Miseikis, T. Siegel, P. Hawkins, A. Harvey, Z. Mašín, S. Patchkovskii, M. Vacher, J. P. Malhado, M. Y. Ivanov, O. Smirnova, J. P. Marangos, Extracting sub-cycle electronic and nuclear dynamics from high harmonic spectra. *Sci. Rep.* **11**, 2485 (2021).

14. M. Vacher, A. Boyer, V. Loriot, F. Lépine, S. Nandi, Few-femtosecond isotope effect in polyatomic molecules ionized by extreme ultraviolet attosecond pulse trains. *J. Phys. Chem.* **126**, 5692–5701 (2022).

15. C. E. M. Gonçalves, R. D. Levine, F. Remacle, Ultrafast geometrical reorganization of a methane cation upon sudden ionization: An isotope effect on electronic non-equilibrium quantum dynamics. *Phys. Chem. Chem. Phys.* **23**, 12051–12059 (2021).

16. S. Patchkovskii, M. S. Schuurman, Full-dimensional treatment of short-time vibronic dynamics in a molecular high-order-harmonic-generation process in methane. *Phys. Rev. A* **96**, 053405 (2017).

17. P. M. Paul, E. S. Toma, P. Breger, G. Mullot, F. Auge, P. Balcou, H. G. Muller, P. Agostini, Observation of a train of attosecond pulses from high harmonic generation. *Science* **292**, 1689–1692 (2001).

18. F. Krausz, M. Ivanov, Attosecond physics. *Rev. Mod. Phys.* **81**, 163–234 (2009).

19. M. Huppert, I. Jordan, D. Baykusheva, A. von Conta, H. J. Wörner, Attosecond delays in molecular photoionization. *Phys. Rev. Lett.* **117**, 093001 (2016).

20. S. Nandi, E. Plésiat, S. Zhong, A. Palacios, D. Busto, M. Isinger, L. Neoričić, C. L. Arnold, R. J. Squibb, R. Feifel, P. Decleva, A. L'Huillier, F. Martín, M. Gisselbrecht, Attosecond timing of electron emission from a molecular shape resonance. *Sci. Adv.* **6**, 7762–7793 (2020).

21. J. Vos, L. Cattaneo, S. Patchkovskii, T. Zimmermann, C. Cirelli, M. Lucchini, A. Kheifets, A. S. Landsman, U. Keller, Orientation-dependent stereo Wigner time delay and electron localization in a small molecule. *Science* **360**, 1326–1330 (2018).

22. H. Ahmadi, E. Plésiat, M. Moioli, F. Frassetto, L. Poletto, P. Decleva, C. D. Schröter, T. Pfeifer, R. Moshammer, A. Palacios, F. Martin, G. Sansone, Attosecond photoionisation time delays reveal the anisotropy of the molecular potential in the recoil frame. *Nat. Commun.* **13**, 1242 (2022).

23. S. Haessler, B. Fabre, J. Higuet, J. Caillat, T. Ruchon, P. Breger, B. Carré, E. Constant, A. Maquet, E. Mével, P. Salières, R. Taïeb, Y. Mairesse, Phase-resolved attosecond near-threshold photoionization of molecular nitrogen. *Phys. Rev. A* **80**, 011404 (2009).

24. L. Cattaneo, J. Vos, R. Y. Bello, A. Palacios, S. Heuser, L. Pedrelli, M. Lucchini, C. Cirelli, F. Martín, U. Keller, Attosecond coupled electron and nuclear dynamics in dissociative ionization of H2. *Nat. Phys.* **14**, 733–738 (2018).

25. L. Cattaneo, L. Pedrelli, R. Y. Bello, A. Palacios, P. D. Keathley, F. Martín, U. Keller, Isolating attosecond electron dynamics in molecules where nuclei move fast. *Phys. Rev. Lett.* **128** (2022).

26. J. W. Rabalais, T. Bergmark, L. O. Werme, L. Karlsson, K. Siegbahn, The jahn-teller effect in the electron spectrum of methane. *Phys. Scr.* **3**, 13–18 (1971).

27. R. Moshammer, M. Unverzagt, W. Schmitt, J. Ullrich, H. Schmidt-Böcking, A 4π recoil-ion electron momentum analyzer: A high-resolution "microscope" for the investigation of the dynamics of atomic, molecular and nuclear reactions. *Nucl. Instrum. Methods Phys. Res. B* **108**, 425–445 (1996).

28. J. Ullrich, R. Moshammer, R. Dörner, O. Jagutzki, V. Mergel, H. Schmidt-Böcking, L. Spielberger, Recoil-ion momentum spectroscopy. *J. Phys. B At. Mol. Opt. Phys.* **30**, 2917–2974 (1997).

29. R. Dörner, V. Mergel, O. Jagutzki, L. Spielberger, J. Ullrich, R. Moshammer, H. Schmidt-Böcking, Cold target recoil ion momentum spectroscopy: A 'momentum microscope' to view atomic collision dynamics. *Phys. Rep.* **330**, 95–192 (2000).

30. J. Ullrich, R. Moshammer, A. Dorn, R. Dörner, L. Ph. H. Schmidt, H. Schmidt-Böcking, Recoil-ion and electron momentum spectroscopy: Reaction-microscopes. *Prog. Phys.* **66**, 1463–1545 (2003).

31. W. A. Chupka, Mass-spectrometric study of the photoionization of methane. *J. Chem. Phys.* **48**, 2337–2341 (1968).

32. C. J. Latimer, R. A. Mackie, A. M. Sands, N. Kouchi, K. F. Dunn, The dissociative photoionization of methane in the VUV. *J. Phys. B At. Mol. Opt. Phys.* **32**, 2667–2676 (1999).

33. T. A. Field, J. H. D. Eland, The fragmentation of CH_4^+ ions from photoionization between 12 and 40 eV. *J. Electron Spectros. Relat. Phenomena* **73**, 209–216 (1995).

34. C. Bourassin-Bouchet, L. Barreau, V. Gruson, J.-F. Hergott, F. Quéré, P. Salières, T. Ruchon, Quantifying decoherence in attosecond metrology. *Phys. Rev. X* **10**, 031048 (2020).

35. S. Patchkovskii, J. Benda, D. Ertel, D. Busto, Theory of nuclear motion in rabbitt spectra. *Phys. Rev. A* **107**, 043105 (2023).

36. J. Benda, Z. Mašín, Multi-photon above threshold ionization of multi-electron atoms and molecules using the R-matrix approach. *Sci. Rep.* **11**, 11686 (2021).

37. J. Benda, Z. Mašín, J. D. Gorfinkiel, Analysis of RABITT time delays using the stationary multiphoton molecular ***R***-matrix approach. *Phys. Rev. A* **105**, 053101 (2022).

38. E. J. Heller, The semiclassical way to molecular spectroscopy. *Acc. Chem. Res.* **14**, 368–375 (1981).

39. T. Mondal, A. J. C. Varandas, Structural evolution of the methane cation in subfemtosecond photodynamics. *J. Chem. Phys.* **143**, 014304 (2015).

40. T. Mondal, A. J. C. Varandas, On extracting subfemtosecond data from femtosecond quantum dynamics calculations: The methane cation. *J. Chem. Theory Comput.* **10**, 3606–3616 (2014).

41. C. Arnold, O. Vendrell, R. Santra, Electronic decoherence following photoionization: Full quantum-dynamical treatment of the influence of nuclear motion. *Phys. Rev. A* **95**, 033425 (2017).

42. M. Ruberti, S. Patchkovskii, V. Averbukh, Quantum coherence in molecular photoionization. *Phys. Chem. Chem. Phys.* **24**, 19673–19686 (2022).

43. B. H. Mahan, A. O'Keefe, Electron impact dissociation of $CH_4(CD_4)$: Laser induced fluorescence of product $CH^+(CD^+)$. *Chem. Phys.* **69**, 35–44 (1982).

44. H. Ahmadi, S. Kellerer, D. Ertel, M. Moioli, M. Reduzzi, P. K. Maroju, A. Jäger, R. N. Shah, J. Lutz, F. Frassetto, L. Poletto, F. Bragheri, R. Osellame, T. Pfeifer, C. D. Schröter, R. Moshammer, G. Sansone, Collinear setup for delay control in two-color attosecond measurements. *J. Phys. Photonics* **2**, 024006 (2020).

45. D. Ertel, M. Schmoll, S. Kellerer, A. Jäger, R. Weissenbilder, M. Moioli, H. Ahmadi, D. Busto, I. Makos, F. Frassetto, L. Poletto, C. D. Schröter, T. Pfeifer, R. Moshammer, G. Sansone, Ultrastable, high-repetition-rate attosecond beamline for time-resolved XUV-IR coincidence spectroscopy. *Rev. Sci. Instrum.* **94**, 073001 (2023).

46. Z. Mašín, J. Benda, J. D. Gorfinkiel, A. G. Harvey, J. Tennyson, Ukrmol+: A suite for modelling electronic processes in molecules interacting with electrons, positrons and photons using the R-matrix method. *Comput. Phys. Commun.* **249**, 107092 (2020).

47. D. G. A. Smith, L. A. Burns, A. C. Simmonett, R. M. Parrish, M. C. Schieber, R. Galvelis, P. Kraus, H. Kruse, R. Di Remigio, A. Alenaizan, A. M. James, S. Lehtola, J. P. Misiewicz, M. Scheurer, R. A. Shaw, J. B. Schriber, Y. Xie, Z. L. Glick, D. A. Sirianni, J. S. O'Brien, J. M. Waldrop, A. Kumar, E. G. Hohenstein, B. P. Pritchard, B. R. Brooks, H. F. Schaefer, A. Y. Sokolov, K. Patkowski, A. E. DePrince, U. Bozkaya, R. A. King, F. A. Evangelista, J. M. Turney, T. D. Crawford, C. D. Sherrill, PSI4 1.4: Open-source software for high-throughput quantum chemistry. *J. Chem. Phys.* **152**, 184108 (2020).

48. D. Ertel, "Attosecond coincidence spectroscopy of methane and deuteromethane," thesis, University of Freiburg (2022).

49. O. Dutuit, M. Aït-Kaci, J. Lemaire, M. Richard-Viard, Dissociative photoionisation of methane and its deuterated compounds in the *A* state region. *Phys. Scr.* **1990**, 223 (**1990**).

50. R. Stockbauer, Threshold electron-photoion coincidence mass spectrometric study of ch4, cd4, c2h6, and c2d6. *J. Chem. Phys.* **58**, 3800–3815 (1973).

51. S. L. Sorensen, A. Karawajczyk, C. Strömholm, M. Kirm, Dissociative photoexcitation of CH_4 and CD_4. *Chem. Phys. Lett.* **232**, 554–560 (1995).

52. T. H. Dunning Jr., Gaussian basis sets for use in correlated molecular calculations. I. The atoms boron through neon and hydrogen. *J. Chem. Phys.* **90**, 1007–1023 (1989).

53. R. A. Kendall, T. H. Dunning Jr., R. J. Harrison, Electron affinities of the first-row atoms revisited. Systematic basis sets and wave functions. *J. Chem. Phys.* **96**, 6796–6806 (1992).

54. M. S. Schuurman, D. R. Yarkony, On the vibronic coupling approximation: A generally applicable approach for determining fully quadratic quasidiabatic coupled electronic state Hamiltonians. *J. Chem. Phys.* **127**, 094104 (2007).

55. S. Patchkovskii, M. S. Schuurman, Short-time dynamics at a conical intersection in high-harmonic spectroscopy. *J. Phys. Chem. A* **118**, 12069–12079 (2014).

56. D. J. Tannor, *Introduction to Quantum Mechanics: A Time-Dependent Perspective* (Univ. Science Books, 2006).

57. U. Jacovella, H. J. Wörner, F. Merkt, Jahn-Teller effect and large-amplitude motion in CH_4^+ studied by high-resolution photoelectron spectroscopy of CH_4. *J. Mol. Spectrosc.* **343**, 62–75 (2018).

58. C. Backx, G. R. Wight, R. R. Tol, M. J. Van der Wiel, Electron-electron coincidence measurements of CH_4. *J. Phys. B At. Mol. Phys.* **8**, 3007–3019 (1975).

59. G. V. Marr, R. M. Holmes, The angular distribution of photoelectrons from CH_4 as a function of photon energy from near threshold to 30 eV. *J. Phys. B* **13**, 939–943 (1980).

60. J. Berkowitz, Polyatomic molecules, in *Atomic and Molecular Photoabsorption*, J. Berkowitz, Ed. (Academic Press, 2015), pp. 391–509.

61. K. Kimura, S. Katsumata, Y. Achiba, T. Yamazaki, S. Iwata, *Handbook of HeI Photoelectron Spectra of Fundamental Organic Molecules* (Japan Scientific Societies Press, 1981).

62. H. Shiromaru, S. Katsumata, Photoelectron angular distribution for Jahn-Teller split bands of some molecules in VUV photoelectron spectroscopy. *Bull. Chem. Soc. Jpn.* **57**, 3543–3551 (1984).

63. M. Stener, G. Fronzoni, D. Toffoli, P. Decleva, Time dependent density functional photoionization of CH4, NH3, H2O and HF. *Chem. Phys.* **282**, 337–351 (2002).

64. S. L. Altmann, P. Herzig, *Point-Group Theory Tables* (Clarendon Press, 1994).

65. F. Gadéa, M. Pélissier, Approximately diabatic states: A relation between effective hamiltonian techniques and explicit cancellation of the derivative coupling. *J. Chem. Phys.* **93**, 545–551 (1990).

66. I. D. Petsalakis, G. Theodorakopoulos, C. A. Nicolaides, R. J. Buenker, Nearly diabatic states by maximization of the non-orthonormal overlap between model-diabatic and mrd-ci wave-functions. *Chem. Phys. Lett.* **185**, 359–364 (1991).

67. T. Pacher, H. Köppel, L. Cederbaum, Quasidiabatic states from ab initio calculations by block diagonalization of the electronic Hamiltonian: Use of frozen orbitals. *J. Chem. Phys.* **95**, 6668–6680 (1991).

68. W. H. Press, S. A. Teukolsky, W. T. Vetterling, B. P. Flannery, *Numerical Recipes in Fortran 77. The Art of Scientific Computing* (Cambridge Univ. Press, ed. 2, 2003).

69. V. I. Lebedev, Values of the nodes and weights of ninth to seventeenth order gauss-markov quadrature formulae invariant under the octahedron group with inversion. *USSR Comput. Math. Math. Phys.* **15**, 44–51 (1975).

70. G. H. Golub, J. H. Welsch, Calculation of gauss quadrature rules. *Math. Comp.* **23**, 221–230 (1969).
